# Three-Dimensional Analysis of Facial Skeleton Textures in CBCT as an Early Warning Sign of Osteoporosis—A Pilot Study

**DOI:** 10.3390/diagnostics16081217

**Published:** 2026-04-19

**Authors:** Tomasz Wach, Marcin Kozakiewicz, Adam Michcik, Marcin Kociołek, Piotr Hadrowicz, Piotr Szymor, Krzysztof Dowgierd, Michał Podgórski, Raphael Olszewski

**Affiliations:** 1 Department of Maxillofacial Surgery, Medical University of Lodz, 251 Pomorska Str., 92-213 Lodz, Poland; marcin.kozakiewicz@umed.lodz.pl (M.K.); piotr.szymor@umed.lodz.pl (P.S.); 2 Department of Maxillofacial Surgery, Medical University of Gdansk, 80-210 Gdańsk, Poland; adammichcik@gumed.edu.pl; 3 Institute of Electronics, Lodz University of Technology, Al. Politechniki 10, 93-590 Łódź, Poland; marcin.kociolek@p.lodz.pl; 4 Department of Otolaryngology, Hospital in Sosnowiec, Zegadłowicza 3, 41-200 Sosnowiec, Poland; phadrowicz@gmail.com; 5 Head and Neck Surgery Clinic for Children and Young Adults, Department of Clinical Pediatrics, University of Warmia and Mazury, ul. Oczapowskiego 2, 10-719 Olsztyn, Poland; krzysztofdowgierd@gmail.com; 6 III Department of Radiology and Diagnostic Imaging, Medical University of Lodz, 90-419 Lodz, Poland; podgorskimt@gmail.com; 7 Department of Oral and Maxillofacial Surgery, Cliniques Universitaires Saint Luc, UCLouvain, 1348 Brussels, Belgium; raphael.olszewski@saintluc.uclouvain.be; 8 Oral and Maxillofacial Surgery Research Lab (OMFSLab), NMSK, Institut de Recherche Expérimentale et Clinique (IREC), UCLouvain, 1348 Brussels, Belgium; 9 Department of Perioperative Dentistry, Ludwika Rydygiera Collegium Medicum in Bydgoszcz, Nicolaus Copernicus University in Torun, 85-067 Torun, Poland

**Keywords:** 3D texture analysis, densitometry, CBCT, CT, bone diagnosis, bone structure, osteoporosis, osteopenia, healthy bone

## Abstract

**Background**: Osteoporosis is a prevalent condition characterized by low bone mass and altered microarchitecture, increasing fracture risk. Early detection remains challenging, as conventional methods such as DXA are limited to specialized settings and often detect disease only after a fracture. Radiomics and three-dimensional (3D) imaging techniques, such as CBCT, may provide novel approaches for assessing bone quality. **Methods**: This pilot study analyzed 68 CBCT scans from adult patients (41 females, 27 males; mean age 57 years). Three-dimensional regions of interest (ROIs) were delineated in seven maxillofacial and mandibular sites (total 309 ROIs). Radiomic texture features were extracted and compared with corresponding T-scores from DXA measurements. Additionally, synthetic 3D reference phantoms with controlled variations in density, trabecular connectivity, and structural anisotropy were generated to evaluate the sensitivity of texture features to microarchitectural changes. **Results**: Several radiomic features, including GLCM-, ARM-, and Gradient-derived parameters, demonstrated consistent monotonic trends correlating with bone density and microstructural deterioration. Differences in feature values were observed across healthy, osteopenic, osteoporotic, and advanced osteoporotic states. Reference phantoms confirmed that the observed trends were attributable to structural differences rather than imaging variability. Features such as Sum Variance and Correlation exhibited potential as early indicators of microarchitectural degradation. **Conclusions**: Three-dimensional CBCT texture analysis may provide a non-invasive, supplementary tool for assessing bone quality and detecting early osteopenic changes. Further studies with larger cohorts are warranted to validate radiomic markers and develop predictive indices for osteoporosis screening.

## 1. Introduction

Osteoporosis is characterized by low bone mass and alterations in bone microarchitecture, which increase bone fragility and the risk of fractures. This common condition primarily affects adult patients and may be diagnosed incidentally or too late, often after a fracture has occurred [1,2].

The literature identifies several risk factors for osteoporosis, including age (>50 years), gender (females are more frequently affected), race, geographic region, genetics, diet, lifestyle factors (e.g., lack of physical activity), and hormonal status. Osteoporosis and associated fractures have become increasingly prevalent due to aging populations [3,4,5,6].

Currently, dual-energy X-ray absorptiometry (DXA) is a widely used standard diagnostic method for assessing bone mineral density (BMD) [4,7]. Additionally, the T-score—a statistical measure used in densitometry that compares an individual’s BMD to the peak bone mass of healthy young adults (20–30 years old)—is commonly used to define bone status: T-score ≥ −1.0 (normal bone), −2.5 < T-score < −1.0 (low bone mass–osteopenia), and T-score ≤ −2.5 (osteoporosis). While DXA is effective and widely available, it has limitations: examinations should be performed on the same DXA device to allow longitudinal comparisons, and patients must be referred to a specialist, often only after suspicion of osteoporosis or post-fracture, when prophylactic intervention may be too late [4,7].

Treatment of diagnosed osteoporosis can be divided into three approaches: pharmacological, non-pharmacological, or a combination of both. Pharmacological treatment includes hormone therapy, bisphosphonates, denosumab, raloxifene, parathyroid hormones, romosozumab, calcium, and vitamin D. Non-pharmacological treatment focuses on lifestyle modification, a balanced diet, adequate calcium and vitamin D intake, exercise, smoking cessation, and avoidance of excessive alcohol consumption [8]. Pharmacological treatment reduces fracture risk by inhibiting bone resorption or stimulating bone formation, while calcium and vitamin D support proper bone mineralization.

Radiomics has recently emerged as a promising—though not yet widely adopted—method for quantitative analysis of radiological images [9,10]. It involves computer-based extraction of radiomic features, which can objectively characterize patterns within tissues, including heterogeneity [10]. “3D texture analysis” is used in the context of radiomics-based extraction and analysis of quantitative texture descriptors from three-dimensional CBCT data.

Maxillofacial and dental surgery clinics routinely perform Cone Beam Computed Tomography (CBCT) and Computed Tomography (CT) for diagnostic and treatment purposes. CBCT/3D imaging constitutes a standard component of the craniomaxillofacial surgical and reconstructive workflow [11,12]. We propose that CBCT imaging could also serve as a supplementary opportunistic screening approach for osteoporosis in settings where it has not yet been explored for this purpose.

This pilot study aims to explore the potential of three-dimensional (3D) texture analysis of maxillofacial bones to assess bone status in humans. Three-dimensional CT performed in dental clinics may provide early indicators of osteoporosis, offering opportunities for earlier diagnosis and intervention.

## 2. Materials and Methods

This pilot study was conducted with the approved by the Bioethics Committee of the Medical University of Łódź (protocol code: RNN/132/25/KE and date of approval: 15 April 2025).

A total of 100 patients who presented to the clinic for consultation were initially considered for inclusion in the study. The availability of a densitometric examination performed in the same patient in the month as CBCT was defined as a key inclusion criterion (T-score of spinal bone density was measured and recorded in the database). Ultimately, 68 CBCT examinations were included in the final analysis (41 females and 27 males; mean age 57 years). Because this was a retrospective pilot study, detailed information on medications affecting bone metabolism and on all systemic comorbidities was not available in a complete and standardized form for all patients.

Three-dimensional regions of interest (ROIs) were manually delineated in seven anatomical locations by only one researcher (TW) in QMaZda software: the anterior maxilla, the posterior (lateral) maxilla, and the maxillary tuberosity; as well as the mandibular symphysis, mandibular body, mandibular ramus, and the head of the mandibular condylar process. In total, 309 ROIs were analysed, with a mean ROI area of 77,844.12 voxels (minimum 1292; maximum 732,924), depending on the anatomical site. The final size of each ROI was influenced, among other factors, by artifacts originating from prosthetic restorations and by interindividual variation in the dimensions of the anatomical regions (e.g., the mandibular ramus represents a relatively narrow structure). No dedicated metal artifact reduction filter or dedicated MAR software was applied during image processing. Instead, ROIs were manually delineated within the trabecular bone while avoiding structures and regions likely to compromise measurement reliability. The ROI was delineated by a single researcher (TW) and the calculated ICC demonstrated high repeatability (>95%) of their measurements (1 researcher; 5 patients; 2 repeats; 7 ROIs for each patient; calculated average for each patient; the repetitions were conducted with a 2-day interval). Each ROI was delineated within the trabecular bone, avoiding the cortical bone, tooth roots, inferior alveolar nerve, maxillary sinus, nasal floor, and incisive canal.

In summary, 128 maxillary regions were analysed (62 in the anterior maxilla, 7 in the posterior maxilla, and 59 in the maxillary tuberosity) and 183 mandibular regions (61 in the mandibular symphysis, 58 in the mandibular body, 39 in the mandibular ramus, and 25 in the mandibular condylar head). Some regions of interest could not be analysed due to limitations in the field of view (FOV) of the CBCT.

The selected ROIs represented trabecular regions of the maxillofacial skeleton that are commonly visible on routine CBCT examinations and may therefore be suitable for opportunistic bone assessment. These sites were chosen to include both maxillary and mandibular regions that are clinically accessible in everyday imaging practice. However, because the number of evaluable ROIs differed across anatomical sites and some regions were limited by field of view, the present pilot study was not designed to establish the superiority of one anatomical location over another.

Artificial intelligence (AI) was used in the study to generate a series of 200 .png files for the construction of each reference standard. A series of digital three-dimensional reference standards was created to systematically investigate how radiomic texture features change in relation to controlled modifications of internal structure, density, and architectural complexity.

All standards were generated computationally within a cubic volume measuring 20 × 20 × 20 mm, discretized into an isotropic voxel grid (voxel size 0.1 mm). This resolution was selected to allow the representation of details comparable to the spatial characteristics of trabecular bone observed in high-resolution CBCT. For each model, a full stack of 200 axial slices was exported in PNG format and subsequently converted into a DICOM image series using the free software 3D Slicer (version 5.2.2). Identical reconstruction parameters were applied to all datasets to ensure that differences in the extracted texture features reflected only the intrinsic structure of the standards.

Each generated volume in DICOM format was then evaluated using RadiAnt DICOM Viewer 2024.1.

**Figure 1:**
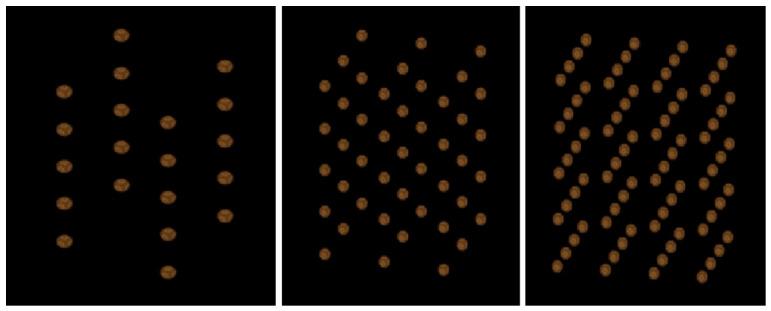
Designed standards obtained by placing 20, 45, and 80 spheres (from left) in an orderly arrangement.

**Figure 2:**
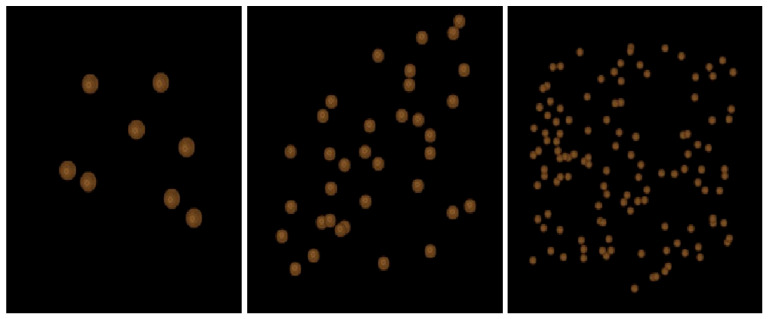
Designed standards obtained by placing spheres in a random distribution with increasing density (from left: sparse, medium, dense).

The study included several categories of reference standards designed to isolate specific structural properties. First, geometric reference models composed of spherical objects were constructed. Three configurations were generated by placing 20, 45, or 80 spheres (diameter 1 mm) uniformly within the cubic volume (Figure 1). Additional spherical models with sparse, medium, and dense spatial distributions were created (assuming that sparse has, e.g., x objects, medium has 2.5x objects, and dense has 4x objects) to investigate the influence of object density on radiomic texture features under strictly controlled conditions. In these models, the variables were the number of objects and their disorder within the 3D space (Figure 2).

**Figure 3:**
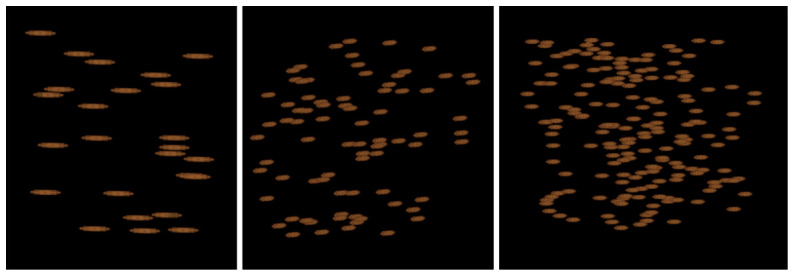
Designed standards by placing ellipsoids in random distribution and increasing density (sparse, medium to dense from left).

Ellipsoidal reference standards with comparable density gradients were also generated to assess whether shape anisotropy affects texture measurements (Figure 3).

**Figure 4:**
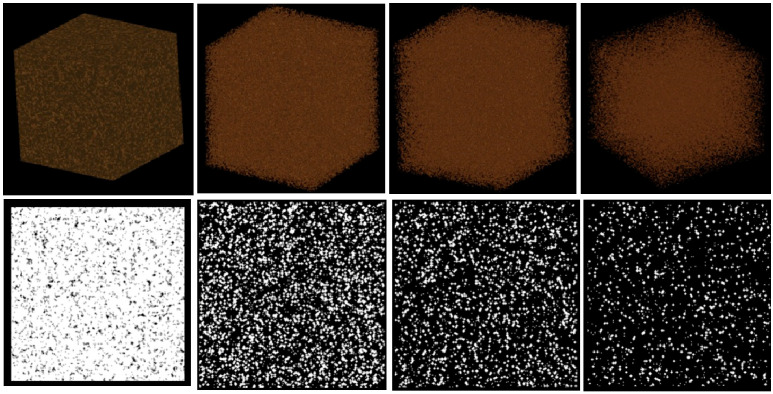
Designed bone standards (from left): D1, D2, D3, and D4. The structures become progressively more trabecular, with decreasing bone density.

To approximate bone-like materials, two groups of trabecular standards were created. The first group mimicked the heterogeneous structure of cancellous bone by generating networks of interconnected trabeculae with densities approximating D1, D2, D3, and D4 bone types (Figure 4). These reference standards were intended to capture the spectrum of bone microarchitecture, from dense and well-organized structures to sparse and mechanically compromised ones.

**Figure 5:**
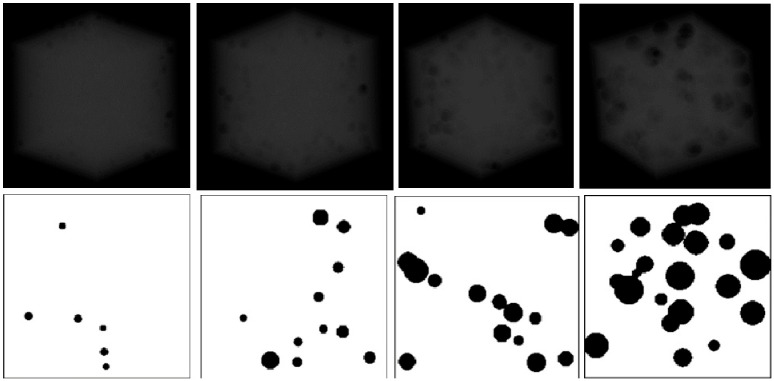
Designed bone standards (from left): healthy bone, osteopenia, osteoporosis, and advanced osteoporosis. Bone density progressively decreases across the models.

Finally, a set of osteoporosis progression models was created to simulate successive stages of trabecular degradation, including a healthy baseline and progressively more degraded states analogous to osteopenia, osteoporosis, and advanced osteoporosis (Figure 5). In these models, trabecular thinning, loss of connectivity, and enlargement of marrow-like void spaces were implemented algorithmically to reflect physiologically plausible patterns of bone loss.

Each phantom was reconstructed in 3D Slicer from the PNG stack, using the same voxel spacing and slice thickness as in the originally generated volume.

Radiomic texture analysis was then performed using the QMaZda software version 20.12 [13]. 

The pixel intensities in all ROIs were normalized and clipped to the range of 0–255 (8-bit), where 0 corresponded to the mean pixel intensity within the ROI minus three standard deviations, and 255 corresponded to the mean plus three standard deviations. For all datasets, a predefined set of features based on the grey-level co-occurrence matrix (GLCM), grey-level run length matrix (GLRM), gradient map, Gabor transform, autoregression model (ARM), and histogram of oriented gradients (HOG) was calculated.

The grey-level co-occurrence matrix (GLCM) is a second-order histogram. It was computed for pairs of points separated by a defined distance on a Cartesian grid and oriented along one of four directions—0°, 45°, 90°, and 135°—in the (x, y) plane, as well as along the direction perpendicular to this plane in the case of three-dimensional ROIs. For each direction and distance, the following features were extracted:

- Angular Second Moment (Energy);
- Contrast;
- Correlation;
- Variance (Sum of Squares);
- Inverse Difference Moment (Homogeneity);
- Sum Average;
- Sum Variance;
- Sum Entropy;
- Entropy;
- Difference Variance;
- Difference Entropy.

For the five directions and five distances, a total of 275 GLCM-based features were calculated.

The gray-level run-length matrix (GRLM) is a two-dimensional matrix p(g,l) that contains the number of runs with a given gray level g and run length l. Similar to the GLCM, it was computed for a total of five directions: 0°, 45°, 90°, and 135° in the (x, y) plane, as well as along the direction perpendicular to this plane. For each direction, the following features were extracted:

- Short Run Emphasis;
- Long Run Emphasis;
- Grey-Level Non-Uniformity;
- Mean Grey-Level Non-Uniformity;
- Run Length Non-Uniformity;
- Mean Run Length Non-Uniformity;
- Fraction.

For the five directions, a total of 35 GRLM-based features were calculated.

The gradient map was computed simultaneously in the vertical and horizontal directions. For this map, the following features were extracted:

- Mean;
- Variance;
- Skewness;
- Kurtosis;
- Non-Zeros.

A total of 5 texture features were extracted.

The autoregressive model provides four regression features accompanied by mean square error.

Gabor (Gab) transform which decomposes image into frequency components was calculated for 6 frequency, standard deviation combinations $\left(\omega ,\sigma \right)$ mainly: (4,2; 6,3; 8,4; 12,6; 16,8; 24,12) and for a total of five directions: 0°, 45°, 90°, and 135° in the (x, y) plane, as well as along the direction perpendicular to this plane. For each such transform average magnitudes were extracted. Total of 30 Gabor transform based features were calculated.

Finally, a Histogram of Oriented Gradients (HOG) was computed for three angular bins (4, 8, and 16), yielding a total of 12 HOG features. Details of all features can be found in the QMaZda manual [14].

The resulting numerical values were compiled into structured datasets that allowed direct comparison of how individual texture features respond to systematic structural changes. This study design enabled the identification of radiomic markers particularly sensitive to trabecular density, connectivity, or the degree of structural degradation. Because all reference standards were generated synthetically under fully controlled conditions, the observed trends in texture features can be attributed exclusively to structural differences rather than imaging variability. This methodological framework provides a reproducible environment for understanding how specific radiomic texture features reflect bone microarchitecture and for assessing their potential utility in diagnosing or monitoring osteopenic and osteoporotic changes in CBCT imaging.

In the subsequent stage, the analysis of the reference standards and the evaluation of changes in texture feature values as a function of the defined 3D structural variables were used to interpret the results of texture feature analysis obtained from real CBCT examinations.

Statistical analysis included evaluation of feature distribution, comparison of means (*t*-test) or medians (W-test), regression analysis, and one-way analysis of variance or the Kruskal–Wallis test, as indicated by non-normal distribution or between-group variance, to assess significant differences among the investigated groups. Differences or relationships were considered statistically significant at *p* < 0.05. Statistical analyses were performed using Statgraphics Centurion version 18.1.12 (StatPoint Technologies, Warrenton, VA, USA). All tests were conducted to evaluate how 3D texture features change in correlation with T-score values and to determine whether any statistically significant correlations exist. Due to the pilot and exploratory nature of the study, no formal correction for multiple comparisons was applied, as the aim of the analysis was to identify potentially relevant radiomic features for further validation rather than to confirm definitive associations. Nevertheless, the increased risk of Type I error should be acknowledged, and the findings should be considered preliminary and requiring confirmation in independent datasets.

## 3. Results

First, analyses were performed for seven maxillofacial regions. A statistically significant (*p* < 0.05) correlation with T-score was identified for 36 of the 399 texture features (Table 1). These 36 texture features were selected for further analysis as an exploratory post hoc feature subset for pilot reporting. The primary analysis was conducted at the patient level, whereas the ROI-level analysis was exploratory and supportive.

Based on the correlation to T-score analysis, a total of 36 texture features were identified that showed a statistically significant relationship with the analyzed dependent variable (*p* < 0.05). Among them, there were 26 features based on the gray-level co-occurrence matrix (GLCM), 5 features from the ARM family (YS8ArmTeta1–4 and YS8ArmSigma), 2 gradient features (YS8GradVariance, YS8GradSkewness), 1 feature based on the Gabor filter (YS8Gab16Z8Mag), and 2 wavelet DWT features (YS8DwtHaarS1HL, YS8DwtHaarS1LH).

The *p*-values for the features included in the table ranged from 0.0009 to 0.0478. The lowest *p*-values were obtained for the features YS8ArmTeta2 (*p* = 0.0009), YS8GradVariance (*p* = 0.0034), YS8GlcmX2DifVarnc (*p* = 0.0047), YS8GlcmX3DifVarnc (*p* = 0.0053), YS8Gab16Z8Mag (*p* = 0.0057), and YS8ArmTeta3 (*p* = 0.0059). The remaining features reached significance at *p* levels between 0.0083 and 0.0478.

**Figure 6:**
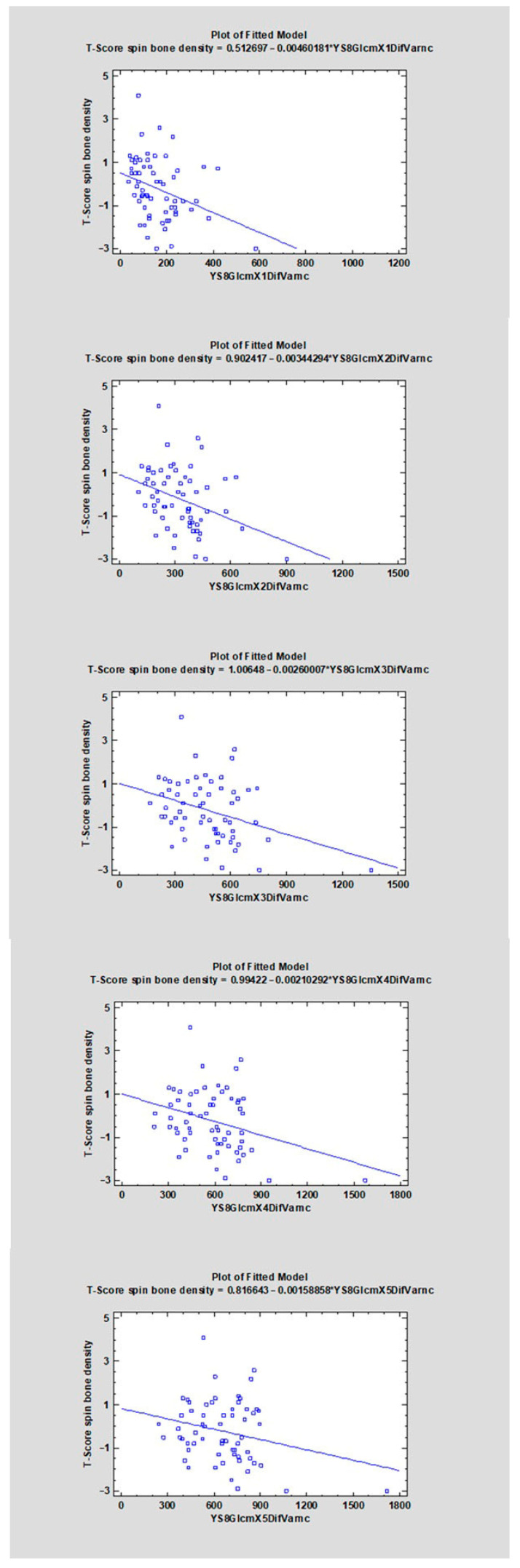
The scatter plot shows the relationship between the variable YS8GlcmX1DifVarnc, YS8GlcmX2DifVarnc, YS8GlcmX3DifVarnc, YS8GlcmX4DifVarnc, YS8GlcmX5DifVarnc (X-axis) and the T-score of spinal bone mineral density (Y-axis). A linear regression model indicating a negative trend—higher values of YS8GlcmX1DifVarnc, YS8GlcmX2DifVarnc, YS8GlcmX3DifVarnc, YS8GlcmX4DifVarnc, YS8GlcmX5DifVarnc are associated with lower T-scores.

**Figure 7:**
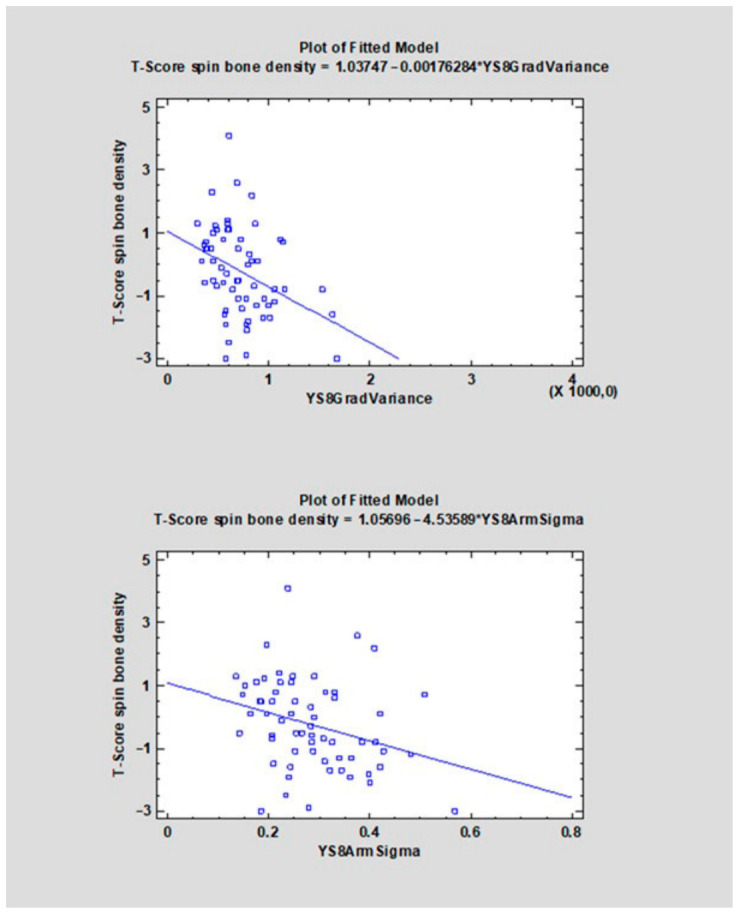
The scatter plot shows the relationship between the variable YS8GradVariance, YS8ArmSigma (X-axis) and the T-score of spinal bone mineral density (Y-axis). A linear regression model indicating a negative trend—higher values of YS8GradVariance, YS8ArmSigma are associated with lower T-scores.

**Figure 8:**
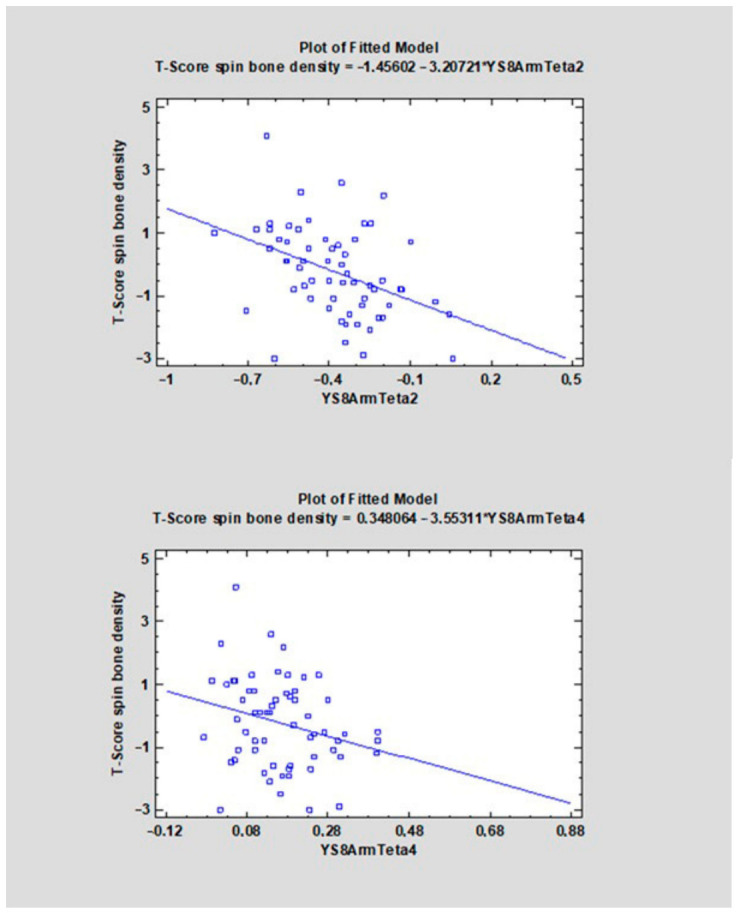
The scatter plot shows the relationship between the variable YS8ArmTeta2, YS8ArmTeta4 (X-axis) and the T-score of spinal bone mineral density (Y-axis). A linear regression model indicating a negative trend—higher values of YS8ArmTeta2 and YS8ArmTeta4 are associated with lower T-scores. YS8ArmTeta2, YS8ArmTeta4 are associated with lower T-scores.

**Figure 9:**
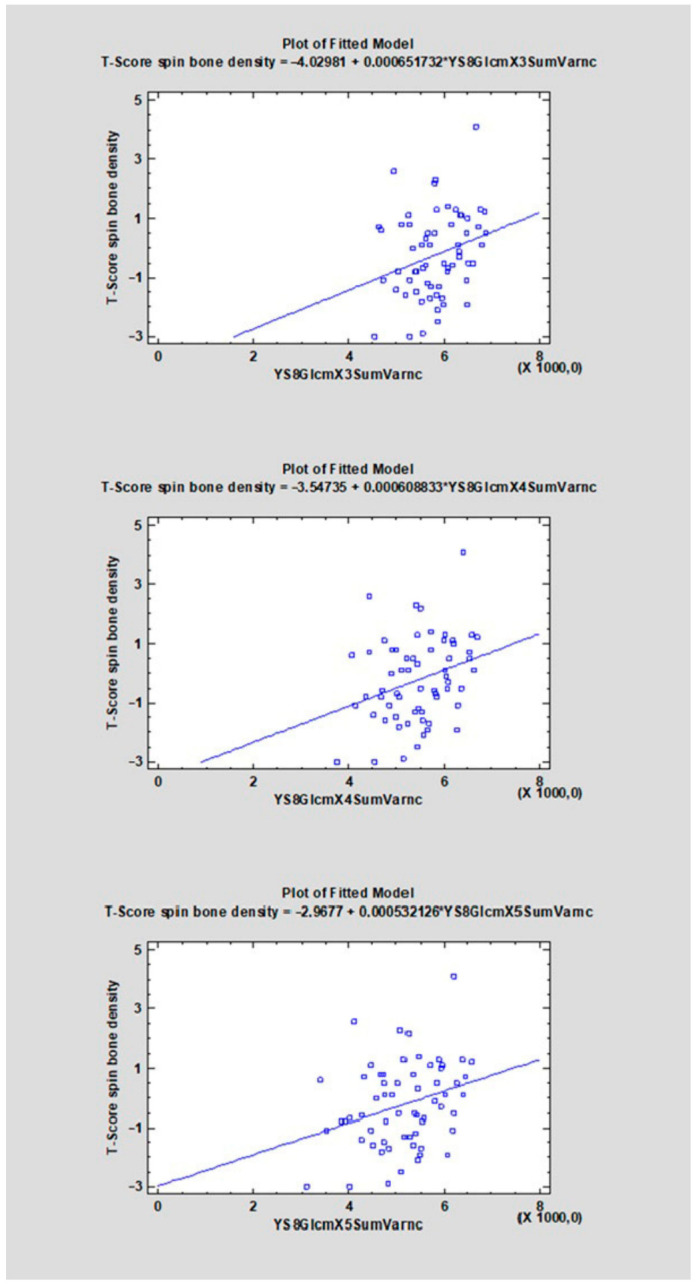
The scatter plot shows the relationship between the variable YS8GlcmX3SumVarnc, YS8GlcmX4SumVarnc, YS8GlcmX5SumVarnc (X-axis) and the T-score of spinal bone mineral density (Y-axis). A linear regression model indicating a positive trend—higher values of YS8GlcmX3SumVarnc, YS8GlcmX4SumVarnc, YS8GlcmX5SumVarnc are associated with higher T-scores.

**Figure 10:**
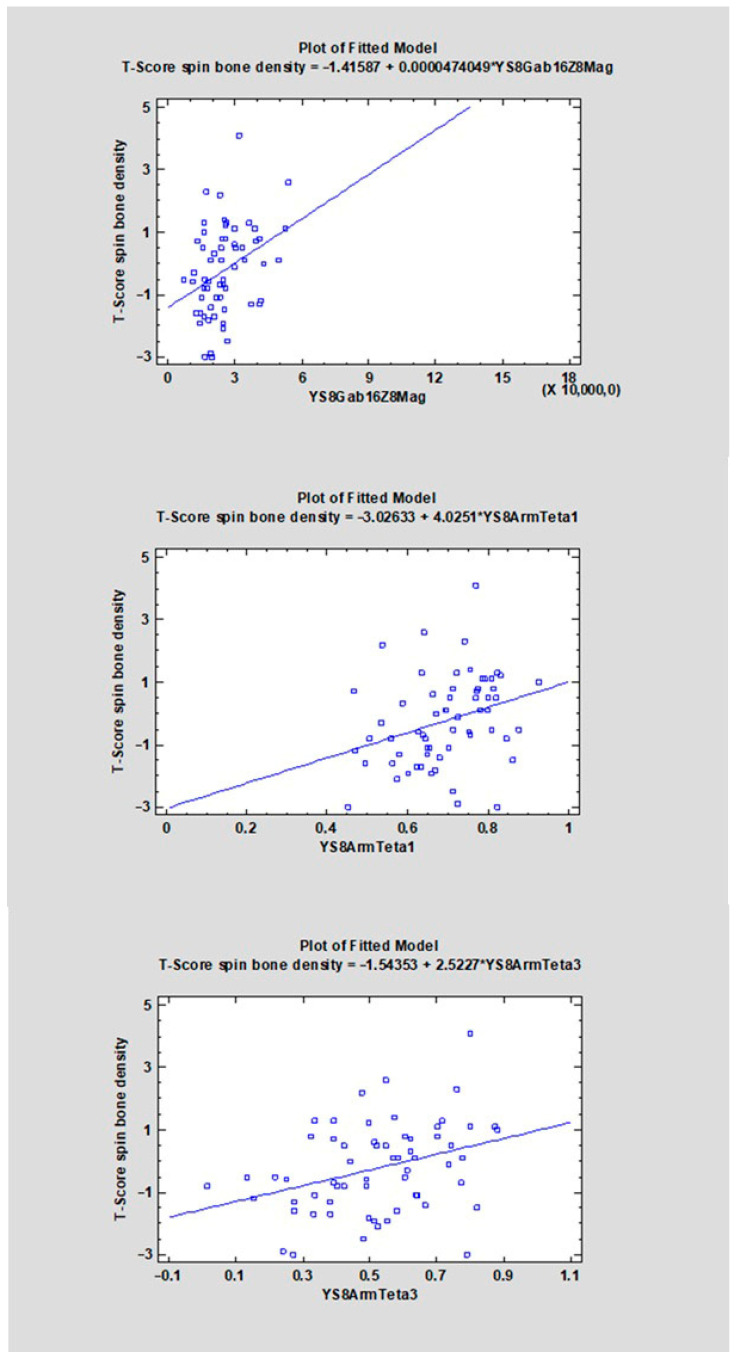
The scatter plot shows the relationship between the variable YS8ArmTeta1, YS8ArmTeta3, YS8Gab16Z8Mag (X-axis) and the T-score of spinal bone mineral density (Y-axis). A linear regression model indicating a positive trend—higher values of YS8ArmTeta1, YS8ArmTeta3, YS8Gab16Z8Mag are associated with higher T-scores.

The correlation coefficients (CC) ranged from −0.4107 to 0.3469 (Table 1). For 22 features, a negative direction of the relationship was found (CC < 0), whereas for 14 features a positive one was observed (CC > 0). The strongest negative correlations with the analyzed variable were observed for YS8ArmTeta2 (CC = −0.4107, R^2^ = 16.87%), followed by YS8GradVariance (CC = −0.3660, R^2^ = 13.40%), YS8GlcmX2DifVarnc (CC = −0.3542, R^2^ = 12.54%), and YS8GlcmX3DifVarnc (CC = −0.3499, R^2^ = 12.24%). The strongest positive correlations were found for YS8Gab16Z8Mag (CC = 0.3469, R^2^ = 12.03%), YS8ArmTeta3 (CC = 0.3458, R^2^ = 11.96%), YS8ArmTeta1 (CC = 0.3113, R^2^ = 9.69%), and YS8GlcmX5SumVarnc (CC = 0.3018, R^2^ = 9.11%) (see Figure 6, Figure 7, Figure 8, Figure 9, Figure 10 and Figures S1–S9).

The R^2^ values for individual texture features ranged from approximately 6.37% to 16.87%, indicating that the full set of analyzed features significantly, although to a varying extent, explained the variability of the studied variable. For features with a positive direction of correlation, R^2^ values were approximately between 6.50% and 12.03%, whereas for features with a negative correlation they ranged from 6.37% to 16.87%.

Additionally, mean values of the statistically significant texture features were calculated for each patient based on the available ROIs. Correlation analysis was then performed between these mean feature values and T-score (Table 2).

Based on the correlation with the analyzed dependent variable, a total of 33 texture features demonstrated a statistically significant relationship (*p* < 0.05) in Table 2. Among them, 25 features were derived from the gray-level co-occurrence matrix (GLCM), 5 belonged to the ARM family (YS8ArmTeta1–4 and YS8ArmSigma), 1 was a gradient-based feature (YS8GradVariance), and 1 represented a wavelet-based DWT parameter (YS8DwtHaarS1LH). The features YS8GradSkewness, YS8Gab16Z8Mag, and YS8DwtHaarS1HL did not reach statistical significance (*p* > 0.05) (Figure S9).

Correlation analysis constituted the principal analytical framework of the present pilot study, as the main objective was to identify radiomic features showing the strongest association with DXA-derived bone status. For this reason, particular attention was given to the direction and strength of the correlations, as expressed by correlation coefficients and R^2^ values.

The *p*-values for the statistically significant features ranged from 0.0008 to 0.0447. The lowest *p*-values were obtained for the features YS8GlcmX4SumVarnc (*p* = 0.0008), YS8GlcmX3SumVarnc (*p* = 0.0009), YS8GlcmH1DifVarnc (*p* = 0.0021), YS8GlcmX5SumVarnc (*p* = 0.0021), YS8GlcmN1DifVarnc (*p* = 0.0032), and YS8ArmSigma (*p* = 0.0033). The remaining significant features achieved *p*-values between 0.0044 and 0.0447.

Among the statistically significant features, the correlation coefficients (CCs) ranged from −0.3744 to 0.4040. For the majority of features, a negative direction of the relationship was observed; however, several parameters showed moderate positive correlations. The strongest negative correlation with the analyzed variable was noted for YS8GlcmH1DifVarnc (CC = −0.3744, R^2^ = 14.01%), followed by YS8GlcmN1DifVarnc (CC = −0.3597, R^2^ = 12.94%) and YS8ArmSigma (CC = −0.3589, R^2^ = 12.88%). In contrast, the highest positive correlation coefficients were obtained for YS8GlcmX4SumVarnc (CC = 0.4040, R^2^ = 16.32%), YS8GlcmX3SumVarnc (CC = 0.4012, R^2^ = 16.10%), and YS8GlcmX5SumVarnc (CC = 0.3740, R^2^ = 13.99%).

Among the statistically significant texture features, the R^2^ values ranged from 6.24% to 16.32%, indicating that the analyzed parameters explained a moderate but meaningful proportion of variability in the studied dependent variable. For features with a positive direction of correlation, R^2^ values ranged from 7.90% to 16.32%, whereas for negatively correlated features they ranged from 6.24% to 14.01%.

In comparison with Table 1, both analyses demonstrate a similar overall strength of associations, with correlation coefficients reaching approximately ±0.40 and maximum R^2^ values around 16–17%. In both datasets, GLCM-based parameters constitute the dominant group of significant features, particularly those describing difference and sum variance. However, Table 1 is characterized by a greater diversity of significant texture classes, including Gabor and both DWT parameters, and shows the single strongest negative correlation (YS8ArmTeta2). In contrast, Table 2 demonstrates greater structural consistency within the GLCM family, especially for SumVarnc parameters, which achieved the highest positive correlation coefficients and R^2^ values, suggesting a more homogeneous and potentially more stable texture-related signal in this analysis.

Statistical analysis was also performed for the four texture features most strongly correlated with T-score and CBCT parameters (kV and mA). In the analyzed group, the mean anode current was 6.52 mA (SD = 2.80; range 2–14 mA), while the mean tube voltage was 97.55 kV (SD = 13.42; range 60–120 kV). The mean values of the texture parameters were as follows: 287.32 (SD = 123.78) for YS8GlcmH1DifVarnc, 5416.67 (SD = 537.90) for YS8GlcmX3SumVarnc, 5024.63 (SD = 582.42) for YS8GlcmX4SumVarnc, and 4692.30 (SD = 638.51) for YS8GlcmX5SumVarnc. The SumVarnc parameters demonstrated a tendency toward negative skewness, whereas YS8GlcmH1DifVarnc showed positive skewness, indicating heterogeneous distribution patterns of these texture features within the study population.

Correlation analysis revealed statistically significant relationships between anode current and all four texture parameters. The strongest associations were observed for YS8GlcmX3SumVarnc (r = 0.5371; *p* < 0.001) and YS8GlcmH1DifVarnc (r = −0.5147; *p* < 0.001). YS8GlcmX4SumVarnc (r = 0.4937; *p* < 0.001) and YS8GlcmX5SumVarnc (r = 0.4360; *p* = 0.0002) also demonstrated moderate correlations. Regression models confirmed the statistical significance of these relationships, with anode current explaining between 19.0% and 29.5% of the variability in the analyzed texture parameters. In contrast, tube voltage (kV) did not show statistically significant associations with any of the evaluated features (*p* > 0.05), and the R^2^ values were negligible (<1%), indicating no measurable effect of voltage on the analyzed texture characteristics.

Secondly, analyses of reference standard were performed and presented in the table to show how the texture analyses changes taking into account differences between them (Table 3).

### 3.1. Spheres 20/45/80

Across 20 → 45 → 80 spheres, a predominantly increasing trend was observed: 24/36 features increased monotonically, 8/36 decreased monotonically, 2/36 remained constant, and 1/36 was non-monotonic. The table includes an intermediate variant (45 spheres, 3 × 3 × 5); therefore, the analysis was conducted using 20/45/80.

The clearest increases were seen in contrast- and variance-related measures:

- GLCM Contrast (YS8GlcmV1Contrast): 6.65544 -> 15.21530 -> 26.19750.
- GLCM Sum Variance (YS8GlcmV1SumVarnc): 77.8608 -> 177.674 -> 305.127.
- Gradient Variance (YS8GradVariance): 37.8806 -> 86.4578 -> 148.518.

Decreases 9/36 were mainly related to GLCM Correlation (e.g., YS8GlcmV1Correlat: 0.842505 -> 0.842238 -> 0.841862) and selected ARM features (YS8ArmTeta1/2/4).

Exceptions and constant features:

- Non-monotonic Gabor magnitude: YS8Gab16Z8Mag 2092.96 -> 3146.23 -> 3055.06 (peak at the intermediate variant).
- Two DWT features remained 0: YS8DwtHaarS1HL and YS8DwtHaarS1LH.

### 3.2. Spherical: Sparse/Medium/Dense

From sparse → medium → dense, local variability and contrast measures increased, whereas several correlation and sum-variance measures decreased. Overall, 19/36 features increased, 15/36 decreased, and 2/36 remained constant.

Examples of increases:

- YS8GlcmV1Contrast: 10.4186 -> 16.2483 -> 24.1054.
- YS8GradVariance: 62.7768 -> 97.8897 -> 141.164.
- YS8ArmSigma: 0.301402 -> 0.380418 -> 0.477411.
- YS8Gab16Z8Mag: 1668.25 -> 1727.42 -> 4893.10 (marked rise in the dense setting).

Examples of decreases:

- YS8GlcmV1SumVarnc: 262.419 -> 248.632 -> 228.157.
- YS8GlcmX4Correlat: 0.706055 -> 0.531835 -> 0.314514.

### 3.3. Ellipsoid: Sparse/Medium/Dense

For ellipsoids, the strongest monotonic increase was observed: 25/36 features increased monotonically, 6/36 decreased monotonically, 3/36 were non-monotonic, and 2/36 remained constant.

Two DWT features (YS8DwtHaarS1HL, YS8DwtHaarS1LH) remained 0.

### 3.4. Bone Like D1/D2/D3/D4

Across the D1–D4 groups, non-monotonic patterns predominated: 17/36 features were non-monotonic, 16/36 increased monotonically, and 3/36 decreased monotonically.

Example of the non-monotonic pattern:

- YS8GlcmH1DifVarnc: 1843.64 -> 1829.39 -> 2055.99 -> 2295.75.

Examples of monotonic increases:

- YS8GlcmV1SumVarnc: 3072.48 -> 4458.60 -> 4574.94 -> 4635.13.
- YS8GlcmV1Correlat: 0.196236 -> 0.250049 -> 0.263753 -> 0.273779.
- YS8Gab16Z8Mag: 12,773.30 -> 15,328.90 -> 15,404.70 -> 15,629.00.

### 3.5. Healthy Bone/Osteopenia/Osteoporosis/Advanced Osteoporosis

In the bone dataset (Healthy → Osteopenia → Osteoporosis → Advanced Osteoporosis), most features increased from the healthy state to osteopenia/osteoporosis but frequently showed a plateau or slight correction in advanced osteoporosis.

Trend distribution was as follows: 12/36 features increased monotonically, 1/36 decreased monotonically, and 23/36 exhibited non-monotonic behavior.

Examples of increases (often “jump + plateau”):

- YS8GlcmV1SumVarnc: 1944.75 -> 4973.57 -> 6980.68 -> 7018.43.
- YS8GlcmV1Correlat: 0.872627 -> 0.908512 -> 0.933672 -> 0.934775.
- YS8GlcmV1Contrast: 132.278 -> 238.416 -> 239.448 -> 236.606.

Clear monotonic decrease:

- YS8ArmSigma: 0.386980 -> 0.329100 -> 0.279615 -> 0.263148.

Selected non-monotonic examples:

- YS8GradVariance: 783.409 -> 1379.38 -> 1380.38 -> 1204.61 (decrease in advanced osteoporosis).
- YS8Gab16Z8Mag: 8311.08 -> 11,598.20 -> 9162.69 -> 10,381.30.

Table 3 reveals repeatable patterns of texture-feature changes across the five groups. In the phantom groups (20/45/80 spheres as well as spherical and ellipsoid sparse/medium/dense configurations), increasing trends dominate for many measures related to GLCM contrast and variance (e.g., features such as Contrast, DifVarnc., and SumVarnc.). Moreover, in spherical layouts, increasing density is accompanied by a clear decrease in Correlat measures (e.g., for directions X4/X5).

In ellipsoids, most features change more consistently (more often monotonically increasing), and decreases in correlation are not as systematic as in spherical phantoms. In the D1–D4 series, non-monotonic trajectories prevail, frequently with a deviation at D2 and subsequent increases toward D3/D4; at the same time, YS8ArmSigma decreases monotonically in this group.

In the bone group (Healthy → Osteopenia → Osteoporosis → Advanced Osteoporosis), many features increase from the healthy state to osteopenia/osteoporosis, whereas advanced osteoporosis often shows a plateau or a minor correction in values. In parallel, a monotonic decrease in YS8ArmSigma is observed.

In addition, the wavelet features YS8DwtHaarS1HL and YS8DwtHaarS1LH are zero in groups 1–3, whereas they are not consistently zero in the D group and in the bone group, which constitutes a clear difference between the phantom and the D/bone datasets.

Based on the table analysis within the bone group (Healthy → Osteopenia → Osteoporosis → Advanced Osteoporosis), several texture features demonstrate consistent monotonic changes across disease stages. The most unambiguous feature is observed among the GLCM-derived measures, namely SumVarnc., which increases throughout all stages. Additionally, this texture feature most strongly “stretches” the scale between healthy bone and osteopenia/osteoporosis; therefore, it appears to be particularly suitable for detecting and tracking changes, especially in the early stages.

**Figure 11:**
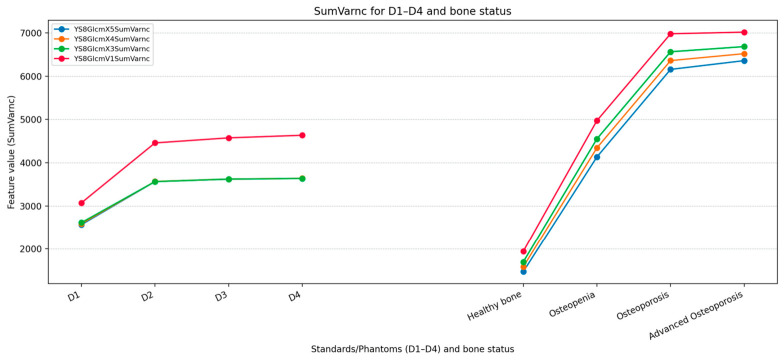
SumVarnc (GLCM sum variance) for four texture features (YS8GlcmX5SumVarnc, YS8GlcmX4SumVarnc, YS8GlcmX3SumVarnc, YS8GlcmV1SumVarnc) across standards D1–D4 and bone-status classes (Healthy, Osteopenia, Osteoporosis, Advanced Osteoporosis).

This descriptor therefore constitutes the most promising candidate presented in the table for further evaluation in detecting bone changes associated with osteopenia and osteoporosis. Nevertheless, further and broader research is required to confirm these findings (Figure 11).

Overall, the most relevant finding of this pilot study is that several radiomic features demonstrated consistent and statistically significant correlations with DXA T-score, while selected phantom models reproduced monotonic trends consistent with progressive structural deterioration. Among the analysed parameters, features such as YS8ArmTeta2, YS8GradVariance, and GLCM-derived Sum Variance appeared particularly informative. Taken together, these findings suggest that CBCT-based 3D texture analysis may capture clinically meaningful aspects of bone microarchitecture and may support opportunistic early identification of patients at increased risk of osteopenic or osteoporotic changes.

To improve readability, selected correlation plots with overlapping or supportive information were moved to the Supplementary Material, while the most representative figures were retained in the main text.

## 4. Discussion

Osteoporosis is a systemic bone disorder characterized by low bone mass and deterioration of bone microarchitecture, leading to increased bone fragility and susceptibility to fractures. According to the NIH, impaired bone strength in this condition significantly increases the risk of fractures, underscoring the importance of both bone quality and density. Osteoporosis is often considered a subclinical condition, with the first clinical manifestation typically being a bone fracture. It is diagnosed at a T-score ≤ −2.5, whereas “severe/established” osteoporosis is defined as a T-score ≤ −2.5 accompanied by a low-energy fracture. Osteopenia (low bone mass) corresponds to a T-score between −1.0 and −2.5, and normal bone mineral density is defined as a T-score ≥ −1.0 [15,16,17,18,19]. The present findings suggest that 3D texture analysis of the maxillofacial region using CBCT may detect differences in bone structure and may help distinguish between healthy and osteoporotic bone. Moreover, texture features derived from 3D CBCT may reflect varying T-score levels, offering the potential not only to distinguish between healthy and osteoporotic bone but also to discriminate among different stages of bone condition, including healthy, osteopenic, osteoporotic, and advanced osteoporotic states.

The uniqueness of this work lies in the development of dedicated models/phantoms, which were subjected to radiological image texture analysis. No comparable solution to this problem has been identified in the scientific literature. By analyzing the proposed models, the authors were able to demonstrate how radiological texture values vary depending on bone quality, while minimizing the risk of error—for example, during ROI delineation. The manufactured phantoms represent an original, modern, and individualized approach aimed at developing an algorithm that could, in the future, facilitate a method for the early detection of bone diseases. One point that may warrant further consideration is that, for D2 bone, radiological texture values initially decrease and then increase. This phenomenon may be explained by the substantially higher density of D1 bone compared with D2, coupled with the very low proportion of trabecular structure in D1—accounting for the observed threshold pattern in the calculated values.

Although the phantom framework confirmed that selected radiomic features respond to controlled changes in density, connectivity, and structural degradation, a direct quantitative comparison between phantom-derived and clinical regression slopes was beyond the scope of this pilot study. This should be addressed in future work to further strengthen the translational interpretation of phantom-to-clinical correspondence.

The research showed that four texture features derived from the grey-level co-occurrence matrix (GLCM), a second-order histogram, were statistically significant: Correlation, Sum Variance, Difference Variance, and Contrast. Two of these features, Correlation and Sum Variance, indicated that higher values correspond to higher T-scores. In contrast, the opposite relationship was observed for Difference Variance and Contrast. In biological terms, GLCM-derived features may reflect local spatial heterogeneity and gray-level transitions within trabecular bone, whereas ARM- and Gradient-derived parameters may capture aspects of structural regularity, directional organization, and local intensity variation associated with microarchitectural deterioration. In this context, features such as Sum Variance and Correlation may be interpreted as indirect descriptors of trabecular complexity and progressive structural disorganization.

Although a comprehensive interpretation of most texture features is lacking, for certain simple textures with well-defined properties, a simplified interpretation of these descriptors may be attempted. The difficulty in interpretability arises from the fact that texture features derived from the grey-level co-occurrence matrix (GLCM) are highly nonlinear. As mentioned earlier, the co-occurrence matrix is a second-order histogram computed for pairs of pixels. It is a two-dimensional matrix in which the column index corresponds to the grey level of the first pixel, and the row index corresponds to the grey level of the second pixel.

**Figure 12:**
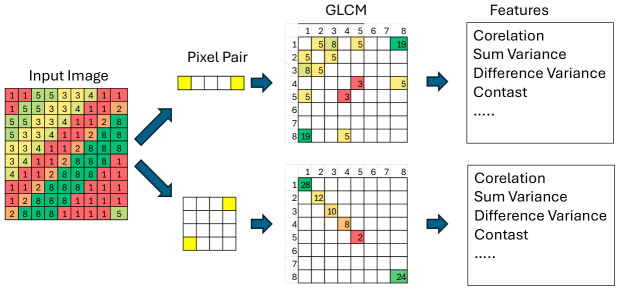
Example of GLCM creation.

The figure below (Figure 12) illustrates a sample image fragment with grey levels ranging from 1 to 8, two pairs of points used to construct this matrix, and the resulting co-occurrence matrices generated for this fragment using the respective pixel pairs.

The content of the co-occurrence matrix depends on multiple factors. First, prior to computing the matrix, the image is normalized to a predefined range of gray levels, typically corresponding to 3 to 8 bits used to encode intensity. Consequently, the size of the co-occurrence matrix usually ranges from 8 × 8 to 256 × 256. It should also be noted that, to maintain consistency with the seminal paper by Haralick, gray-level values are indexed starting from 1. Furthermore, depending on the image content, the distance between test pixels, and the orientation along which the pixel pairs are defined, the co-occurrence matrix will take different forms. For a smooth texture with a single gray level, the resulting co-occurrence matrix will contain a single non-zero value located on the diagonal at the intersection corresponding to the gray level of the pixels. For a directional texture, a co-occurrence matrix computed in the direction aligned with the dominant orientation of structures in the image will exhibit a predominantly diagonal pattern. However, when computed in a direction misaligned with the image structures, the non-zero elements will appear farther from the diagonal.

If the structures in the image exhibit circular symmetry, then for a given pixel-pair distance the co-occurrence matrix will be invariant with respect to the direction along which the test pixels are positioned.

In a highly simplified form, the following interpretations can be stated:

**Correlation** in the GLCM measures the degree of linear dependency between the intensities of pixels and their neighbors. In other words, it evaluates whether pixel brightness changes in a predictable manner along a given direction.

**Contrast** quantifies the differences in intensity between pixels—how strong these differences are and how frequently they occur. In practice, it provides information about the “roughness” and the local contrast of the texture.

**Sum Variance** measures the variability (spread) of pixel intensities in the image but weighted by the sums of the gray-level pairs (i + j). It is therefore a measure of contrast and textural complexity, but distinct from “Contrast” as it is more sensitive to tonal variations throughout the entire texture structure.

**Difference Variance** measures the variability (variance) of the distribution of intensity differences between pixel pairs. It is based on the so-called difference histogram, which analyzes values |i − j| instead of the gray levels themselves. It quantifies how much intensity differences between neighboring pixels vary. In practice, it is a measure of heterogeneity, roughness, and local variability of the texture.

For the example image discussed earlier, when the direction of the GLCM is aligned with the dominant orientation of the texture, the Correlation, Difference Variance, and Sum Variance will be close to zero. The opposite will hold for a direction misaligned with the image orientation. In the general case, however, the interpretation of these features becomes more complex.

The present study identified Difference Variance and Contrast as statistically significant texture features associated with T-scores. In the phantoms used in this research, variations in these parameters were linked to changes in object structure, indicating sensitivity to differences in structural organization. In particular, their behaviour appeared to reflect increasing heterogeneity, disorganization, and architectural complexity within the analysed structures. Furthermore, these features varied across bone types (D1, D2, D3, D4) and bone conditions (healthy, osteopenic, osteoporotic), corresponding to progressive deterioration in bone quality observed both from D1 to D4 and from healthy bone to advanced osteoporosis. By contrast, Correlation and Sum Variance demonstrated an opposing pattern of association (Figure 6 and Figures S1–S8), suggesting that individual texture parameters may capture different aspects of bone microarchitecture. Taken together, these findings indicate that the analysed texture features, through their characteristic variation, may serve as markers of differences in bone quality and overall skeletal health. As bone structure becomes increasingly porous and affected by osteoporotic changes, its microarchitectural organization is altered, and the 3D texture analysis approach applied in the present study appears capable of effectively capturing these alterations [2,20].

Bone mineral density (BMD) measurement forms the basis for diagnosis and risk assessment, as there are currently no satisfactory clinical methods for evaluating “bone quality.” Therefore, in clinical practice, diagnosis is primarily based on quantitative measurement of bone mass, most commonly using the DXA method. In classic densitometric assessment, the recommended site for DXA testing is the proximal femur; central DXA of the lumbar spine and hip is also routinely performed, while forearm measurement is indicated in specific clinical situations (e.g., when the hip or spine cannot be reliably assessed). The FRAX tool calculates the 10-year probability of fracture—including hip and “major osteoporotic fracture”—based on clinical risk factors, with the option to include or omit femoral neck BMD. Explicitly mentioned risk factors include BMI, prior fracture, parental hip fracture, oral glucocorticoid use, rheumatoid arthritis, smoking, and alcohol consumption. BMD (DXA) testing is indicated in women ≥ 65 years of age, men ≥ 70 years of age, adults following low-energy fractures, and individuals with significant risk factors, as well as in situations where the results influence therapeutic decisions and subsequent monitoring [15,16,18,19,21,22,23]. This research offers promising potential for indicating and diagnosing osteopenia or osteoporosis before incidental fractures occur. Most patients diagnosed with osteoporosis are over 65 years of age or have already experienced a bone fracture. The analysis of texture features presented in this study, routinely performed using maxillofacial CBCT, provides hope for the early detection of osteopenic or osteoporotic changes in the human skeleton. 

Very often, treatment of this condition involves bisphosphonates. This type of pharmacological therapy may be associated with MRONJ (Medication-Related Osteonecrosis of the Jaw), which can lead to jaw fractures following dental procedures; therefore, patients should be appropriately prepared for such treatment [1,8,17,19]. The proposed method for early detection of bone changes may facilitate the management of osteopenia and the prevention of osteoporosis through interventions such as vitamin D and calcium supplementation, as well as lifestyle modifications.

Taking into account parameters of CBCT, it should be emphasized, however, that the present analysis was conducted in a relatively limited patient cohort and within a defined number of ROIs. Although the observed relationships reached statistical significance, they require validation in a larger population and with a greater number of analysed regions of interest to assess their robustness and reproducibility. Moreover, considering the known sensitivity of radiomic features to acquisition parameters and hardware-related factors, future investigations should ideally be performed using a single imaging device. Standardization of both the scanner and acquisition protocol would reduce inter-device variability and allow for a more reliable assessment of the true impact of exposure parameters on texture feature values.

Our findings should also be interpreted in the broader context of opportunistic imaging-based bone assessment. Recent CT-based studies in adult and paediatric liver transplant recipients have shown that routine abdominal CT (and also thoracic and lumbar spine CT) may provide useful supplementary information on bone health, supporting the growing role of opportunistic imaging in skeletal assessment [24,25,26,27].

The main limitation of this study was the small sample size. Further research involving a substantially larger patient cohort of 600 patients is planned and already underway. Several of the top-ranked radiomic features showed moderate correlations with tube current (mA), suggesting a possible influence of acquisition-related variability. From a radiomics perspective, this suggests that tube current may act as a potential confounder, as part of the observed feature variation may reflect acquisition-related differences rather than biological variation alone. This further supports the need for scanner harmonization and protocol standardization in future studies. In addition, detailed scanner model information was not available for all examinations in this retrospective dataset, which prevented a systematic assessment of scanner-related effects. This should be regarded as an important limitation, and the results should therefore be interpreted with caution. Inter-observer variability was also not assessed, so the robustness of ROI delineation across different readers remains uncertain and should be examined in future studies. Site-specific comparison likewise remains an important objective for subsequent investigations with larger and more balanced anatomical sampling. From a practical point of view, the most suitable regions for future clinical implementation are likely to be those that are consistently included in routine CBCT examinations and are relatively easy to delineate within trabecular bone. However, dedicated site-specific validation will be necessary before any anatomical region can be recommended as the preferred screening location. Moreover, despite intensity normalization, residual variability related to scanner model, acquisition settings, and reconstruction parameters cannot be excluded. The present pilot analysis also did not include multivariable adjustment for age and sex, although both are major determinants of bone density; this should be addressed in future larger studies incorporating clinical covariates and more robust multivariable modelling. Finally, given the limited sample size and exploratory pilot design, deriving preliminary diagnostic cut-off values was considered premature. Future validation studies should therefore assess the diagnostic performance of the most informative features, including ROC analysis, sensitivity, specificity, and threshold optimization.

## 5. Conclusions

Thanks to emerging technologies that are increasingly being applied in medicine, many diseases warrant a renewed perspective and a reassessment of existing diagnostic approaches. Osteoporosis and osteopenia constitute relevant examples in this context. The presented pilot study suggests that it may be possible to detect early changes in bone tissue using alternative imaging and analytical techniques.

The proposed approach may contribute to earlier identification of bone alterations using CBCT/CT examinations that are already routinely performed for other clinical indications. In practice, this approach may create an opportunity to identify patients who could be at increased risk of osteoporosis, without the need to introduce additional dedicated diagnostic procedures. However, further validation is required before any clinical application can be considered.

Ongoing studies involving a substantially larger patient cohort are intended to further explore these preliminary findings and to assess whether it is feasible to develop and validate a novel osteoporotic/osteopenic index. In future studies, the most informative features identified in the present pilot analysis may serve as candidate variables for the development of a multivariable predictive model for osteopenic and osteoporotic risk stratification. If validated, such an index could support risk stratification and improve referral for further diagnostic evaluation and treatment. Approval from the institutional bioethics committee has already been obtained for the prospective study.

Therefore, CBCT-based 3D texture analysis should currently be viewed as a potential supplementary tool for bone quality assessment and early risk stratification, rather than as a stand-alone diagnostic method for osteoporosis.

## Figures and Tables

**Table 1:** Table presents the results of the statistical analysis of texture features evaluated for correlation with T-score. A *p*-value < 0.05 was considered statistically significant. Abbreviation: CC, correlation coefficient.

Texture Feature	*p* Value	CC	R-Squared (%)
YS8GlcmH1DifVarnc	0.0338	−0.270048	7.29
YS8GlcmV1Contrast	0.0213	−0.29193	8.52
YS8GlcmV1Correlat	0.0231	0.288143	8.30
YS8GlcmV1SumVarnc	0.0381	0.264	6.97
YS8GlcmV1DifVarnc	0.0095	−0.326908	10.69
YS8GlcmV2DifVarnc	0.0308	−0.274571	7.54
YS8GlcmN1DifVarnc	0.027	−0.280845	7.89
YS8GlcmZ1SumVarnc	0.0443	0.256361	6.57
YS8GlcmZ1DifVarnc	0.0185	−0.298481	8.91
YS8GlcmZ2DifVarnc	0.0434	−0.257475	6.63
YS8GlcmX1Contrast	0.0221	−0.290344	8.43
YS8GlcmX1Correlat	0.0302	0.275564	7.59
YS8GlcmX1DifVarnc	0.0083	−0.33254	11.06
YS8GlcmX2Contrast	0.0255	−0.283619	8.04
YS8GlcmX2Correlat	0.0413	0.260021	6.76
YS8GlcmX2DifVarnc	0.0047	−0.354166	12.54
YS8GlcmX3Contrast	0.0293	−0.276967	7.67
YS8GlcmX3Correlat	0.0372	0.265304	7.04
YS8GlcmX3SumVarnc	0.0328	0.271517	7.37
YS8GlcmX3DifVarnc	0.0053	−0.349892	12.24
YS8GlcmX4Contrast	0.0455	−0.254944	6.50
YS8GlcmX4Correlat	0.0425	0.258491	6.68
YS8GlcmX4SumVarnc	0.0181	0.29938	8.96
YS8GlcmX4DifVarnc	0.0124	−0.315952	9.98
YS8GlcmX5SumVarnc	0.0171	0.301777	9.11
YS8GlcmX5DifVarnc	0.0478	−0.252409	6.37
YS8ArmTeta1	0.0138	0.3113	9.69
YS8ArmTeta2	0.0009	−0.410723	16.87
YS8ArmTeta3	0.0059	0.345825	11.96
YS8ArmTeta4	0.0477	−0.252463	6.37
YS8ArmSigma	0.0171	−0.301952	9.12
YS8GradVariance	0.0034	−0.366027	13.40
YS8GradSkewness	0.0455	0.254999	6.50
YS8Gab16Z8Mag	0.0057	0.346896	12.03
YS8DwtHaarS1HL	0.0455	−0.314165	9.87
YS8DwtHaarS1LH	0.0372	−0.274218	7.52

**Table 2:** Results of the statistical analysis of texture features based on mean patient-level values, evaluated for correlation with T-score. A *p*-value < 0.05 was considered statistically significant. Abbreviation: CC, correlation coefficient; * *p* > 0.05.

Texture Feature	*p* Value	CC	R^2^ (%)
YS8GlcmH1DifVarnc	0.0021	−0.374363	14.01
YS8GlcmV1Contrast	0.005	−0.34404	11.84
YS8GlcmV1Correlat	0.0049	−0.34502	11.90
YS8GlcmV1SumVarnc	0.0068	0.332677	11.07
YS8GlcmV1DifVarnc	0.0044	−0.348515	12.15
YS8GlcmV2DifVarnc	0.0064	−0.335016	11.22
YS8GlcmN1DifVarnc	0.0032	−0.359732	12.94
YS8GlcmZ1SumVarnc	0.0157	−0.298553	8.91
YS8GlcmZ1DifVarnc	0.0093	−0.320078	10.25
YS8GlcmZ2DifVarnc	0.0096	−0.31904	10.18
YS8GlcmX1Contrast	0.019	−0.290327	8.43
YS8GlcmX1Correlat	0.018	0.292578	8.56
YS8GlcmX1DifVarnc	0.0098	−0.318124	10.12
YS8GlcmX2Contrast	0.0209	−0.286132	8.19
YS8GlcmX2Correlat	0.0132	0.305808	9.35
YS8GlcmX2DifVarnc	0.0067	−0.332878	11.08
YS8GlcmX3Contrast	0.0307	−0.268262	7.20
YS8GlcmX3Correlat	0.0110	0.313541	9.83
YS8GlcmX3SumVarnc	0.0009	0.401185	16.10
YS8GlcmX3DifVarnc	0.0071	−0.330984	10.96
YS8GlcmX4Contrast	0.0447	−0.249829	6.24
YS8GlcmX4Correlat	0.0088	−0.322626	10.41
YS8GlcmX4SumVarnc	0.0008	0.404003	16.32
YS8GlcmX4DifVarnc	0.0128	−0.307287	9.44
YS8GlcmX5SumVarnc	0.0021	0.373991	13.99
YS8GlcmX5DifVarnc	0.0318	−0.266676	7.11
YS8ArmTeta1	0.0239	−0.280018	7.84
YS8ArmTeta2	0.0252	−0.277534	7.70
YS8ArmTeta3	0.0094	0.319816	10.23
YS8ArmTeta4	0.0233	0.281072	7.90
YS8ArmSigma	0.0033	−0.358873	12.88
YS8GradVariance	0.0084	−0.324405	10.52
YS8GradSkewness	0.1444 *	0.183045	3.35
YS8Gab16Z8Mag	0.5914 *	−0.0678289	0.46
YS8DwtHaarS1HL	0.2202 *	−0.154145	2.38
YS8DwtHaarS1LH	0.0161	−0.297545	8.85

**Table 3:** Table presents values of texture features for five groups phantom objects. Grey color—increasing values; Yellow color (y)—decreasing values; Blue color (z)—zero; * *p* < 0.001.

	20 Spheres (2 × 2 × 5)	45 Spheres (3 × 3 × 5)	80 Spheres (4 × 4 × 5)	Spherical Sparse	Spherical Medium	Spherical Dense	Ellipsoid Sparse	Ellipsoid Medium	Ellipsoid Dense	D1	D2	D3	D4	Healthy Bone	Osteopenia	Osteoporosis	Advanced Osteoporosis
YS8GlcmH1DifVarnc	6.6526	15.2007	26.1529	10.4117	16.2314	24.103	3.02578	9.36129	20.9556	1843.64	1829.39	2055.99	2295.75	131.184	235.172	235.11	234.139(y)
YS8GlcmV1Contrast	6.65544	15.2153	26.1975	10.4186	16.2483	24.1054	14.2969	34.6617	65.8394	2064.43	2674.88	2665.31	2642.63	132.278	238.416	239.448	236.606(y)
YS8GlcmV1Correlat	0.842505(y)	0.842238(y)	0.841862(y)	0.923628(y)	0.877316(y)	0.808886(y)	0.75621(y)	0.759287(y)	0.74667(y)	0.196236	0.250049	0.263753	0.273779	0.872627	0.908512	0.933672	0.934775
YS8GlcmV1SumVarnc	77.8608	177.674	305.127	262.419(y)	248.632(y)	228.157(y)	102.992	253.33	453.953	3072.48	4458.6	4574.94	4635.13	1944.75	4973.57	6980.68	7018.43
YS8GlcmV1DifVarnc	6.6526	15.2007	26.1529	10.4117	16.2314	24.0682	14.2841	34.5848	65.5575	1843.85	1829.54(y)	2056.26	2296.3	131.318	235.474	235.187(y)	230.403(y)
YS8GlcmV2DifVarnc	12.3783	28.2704	48.608	20.5186	32.0172	46.3131	27.4342	66.2981	122.068	2066.17	1973.19(y)	2299.29	2653.05	259.059	458.734	462.848	406.16(y)
YS8GlcmN1DifVarnc	8.99659	20.5527	35.3516	14.6863	23.2337	33.0732	14.3569	36.031	65.8764	1842.79	1829.32(y)	2055.67	2293.02	185.263	329.117	323.705	344.678
YS8GlcmZ1SumVarnc	75.9451	173.291	297.572	259.524(y)	242.957(y)	220.392(y)	103.516	253.282	456.24	3066.9	4457.65	4577.05	4637.88	1900.52	4899.92	6909.11	6881.4(y)
YS8GlcmZ1DifVarnc	8.99659	20.5527	35.3516	14.6863	23.2337	33.0732	14.3569	36.0885	65.8845	1846.81	1829.85(y)	2055.4	2294.48	184.499	329.995	330.642	340.304
YS8GlcmZ2DifVarnc	17.668	40.3345	69.3095	29.1786	46.0008	65.5771	28.771	70.2789	130.505	2172.28	2064.2(y)	2496.91	2982.59	363.797	646.881	645.419	573.716(y)
YS8GlcmX1Contrast	6.65544	15.2153	26.1975	10.4186	16.2483	24.0664	14.2969	34.674	65.799	2063.75	2674.92	2664.24	2642.67	132.039	238.193	237.47(y)	236.078(y)
YS8GlcmX1Correlat	0.842505(y)	0.842238(y)	0.841862(y)	0.923628(y)	0.877316(y)	0.809131(y)	0.75621(y)	0.759208(y)	0.746834(y)	0.196557	0.250016	0.264077	0.273695	0.872828	0.908591	0.934219	0.934922
YS8GlcmX1DifVarnc	6.6526	15.2007	26.1529	10.4117	16.2314	24.0293	14.2841	34.5971	65.5174	1843.31	1829.55(y)	2055.69	2296.33	131.082	235.256	233.279(y)	229.902(y)
YS8GlcmX2Contrast	12.3881	28.3209	48.7627	20.5477	32.0831	46.3314	27.4818	67.1166	123.418	2352.47	3134.36	3152.17	3146.79(y)	262.01	469.846	475.751	424.884(y)
YS8GlcmX2Correlat	0.708341(y)	0.707843(y)	0.707144(y)	0.850143(y)	0.758981(y)	0.633965(y)	0.533769(y)	0.536272(y)	0.527241(y)	0.084112	0.121181(y)	0.129383	0.135137	0.748632	0.820448	0.868748	0.883204
YS8GlcmX2DifVarnc	12.3783	28.2704	48.608	20.5207	32.0172	46.1941	27.4342	66.8283	122.428	2066.04	1973.65(y)	2300.3	2655.7	258.243	458.421	458.93	404.881(y)
YS8GlcmX3Contrast	18.1798	41.5617	71.5605	30.7812	48.0064	68.7098	40.8026	99.6342	180.933	2522.72	3568.77	3619.45	3640.26	391.832	702.051	713.527	611.91(y)
YS8GlcmX3Correlat	0.574175(y)	0.573444(y)	0.572417(y)	0.776655(y)	0.640996(y)	0.458785(y)	0.311324(y)	0.314434(y)	0.308796(y)	0.0178169(y)	−0.000608719(y)	0.000415452(y)	−0.000394009(y)	0.625252	0.732773	0.803929	0.83225
YS8GlcmX3SumVarnc	67.2065	153.31	263.161	244.857(y)	219.436(y)	185.2(y)	77.6931	191.028	342.598	2614.25	3564.43	3622.46	3637.39	1699.34	4552.29	6564.71	6683.59
YS8GlcmX3DifVarnc	18.1587	41.4529	71.2274	30.7206	47.8589	68.4077	40.6977	98.9989	178.804	2193.33	2064.03(y)	2496.3	2983.08	383.408	676.541	675.691(y)	570.421(y)
YS8GlcmX4Contrast	24.0316	54.9397	94.5945	40.7202	62.8597	87.2293	50.1812	122.321	220.873	2545.96	3565.62	3619.22	3639.82	508.994	929.865	945.701	794.147(y)
YS8GlcmX4Correlat	0.440007(y)	0.439041(y)	0.437684(y)	0.706055(y)	0.531835(y)	0.314514(y)	0.157357	0.160651	0.157268(y)	0.0087411(y)	0.000222879(y)	0.000485214(y)	−0.000180202(y)	0.514365	0.647376	0.741129	0.782866
YS8GlcmX4SumVarnc	61.7966	140.938	241.851	236.34(y)	205.677(y)	167.274(y)	68.9231	169.146	303.31	2590.86	3567.21	3622.73	3638.5	1587.2	4344.11	6360.65	6520.67
YS8GlcmX4DifVarnc	23.9946	54.7496	94.0126	40.6141	62.6069	86.7423	50.0226	121.364	217.7	2210.47	2063.54(y)	2496.21	2982.79	494.778	885.113	879.236(y)	724.267(y)
YS8GlcmX5SumVarnc	56.3302	128.438	220.32	227.732(y)	191.733(y)	149.103(y)	59.948	147.157	263.618	2568.77	3565.99	3620.53	3637.95	1474.14	4134.79	6157.28	6359.68
YS8GlcmX5DifVarnc	29.8869	68.1619	116.965	50.5958	77.4383	105.163	59.1838	143.617	256.487	2227.15	2063.7(y)	2496.72	2982.92	604.044	1088.1	1073.13(y)	868.83(y)
YS8ArmTeta1	0.476651(y)	0.476637(y)	0.476618(y)	0.512312(y)	0.525284(y)	0.487222(y)	0.86544(y)	0.835888(y)	0.791659(y)	0.141986	0.183683	0.192532	0.199116	0.516288(y)	0.51171(y)	0.507625(y)	0.597418
YS8ArmTeta2	−0.02234(y)	−0.02235(y)	−0.02237(y)	−0.0702776(y)	−0.098075 (y)	−0.0381756(y)	0.0432097(y)	−0.068535(y)	0.074561	0.133798	0.150587	0.155039	0.15901	−0.075487	−0.067062	−0.051297	−0.105808(y)
YS8ArmTeta3	0.268929	0.26893	0.268931(y)	0.310019(y)	0.329467(y)	0.275985(y)	−0.132201(y)	0.0583294(y)	−0.11555(y)	0.111614	0.115832	0.11561(y)	0.114877(y)	0.298447	0.30218	0.288941(y)	0.290943
YS8ArmTeta4	0.257292(y)	0.257274(y)	0.257249(y)	0.239416	0.228453	0.252062	0.203476(y)	0.14766(y)	0.22031	0.155604	0.202476	0.213559	0.221752	0.247465(y)	0.243423(y)	0.248957 *	0.212197(y)
YS8ArmSigma	0.435392(y)	0.435759(y)	0.436274(y)	0.301402	0.380418	0.477411	0.306712	0.341488	0.374487	0.945006(y)	0.91475(y)	0.906339(y)	0.899774(y)	0.38698(y)	0.3291(y)	0.279615(y)	0.263148(y)
YS8GradVariance	37.8806	86.4578	148.518	62.7768	97.8897	141.164	61.7115	153.28	287.633	5341.68(y)	3528.57	4961.76	6729.13	783.409	1379.38	1380.38	1204.61(y)
YS8GradSkewness	−28.7578	−19.1285	−14.3008	−22.528	−17.744	−14.9633	−20.1411	−12.6258	−9.3542	−1.34014(y)	0.318883 *	−0.229604(y)	−1.04(y)	−6.6346	−4.97545	−4.07267	−3.32133
YS8Gab16Z8Mag	2092.96(y)	3146.23(y)	3055.06(y)	1668.25	1727.42	4893.1	1427.46	1915.04	2740.55	12,773.3	15,328.9	15,404.7	15,629	8311.08	11,598.2	9162.69(y)	10,381.3
YS8DwtHaarS1HL	0(z)	0(z)	0(z)	0(z)	0(z)	0(z)	0(z)	0(z)	0(z)	293.718	335.004	335.2	330.678(y)	0(z)	2.76618(y)	2.62703(y)	0(z)
YS8DwtHaarS1LH	0(z)	0(z)	0(z)	0(z)	0(z)	0(z)	0(z)	0(z)	0(z)	269.954	329.936	324.118(y)	330.547	1.42324	2.76618	2.4519(y)	0(z)

## Data Availability

The original contributions presented in this study are included in the article. Further inquiries can be directed to the corresponding author.

## Supplementary Materials

The following supporting information can be downloaded at: https://www.mdpi.com/article/10.3390/diagnostics16081217/s1.
