# Supplementary material for: Three-Dimensional Analysis of Facial Skeleton Textures in CBCT as an Early Warning Sign of Osteoporosis—A Pilot Study
Source: Diagnostics (Basel). 2026 Apr 19;16(8):1217. doi: 10.3390/diagnostics16081217 (PMC13114353; doi:10.3390/diagnostics16081217)
Supplement: Supplementary file 1 [file diagnostics-16-01217-s001.zip › diagnostics-4217907-supplementary.pdf]

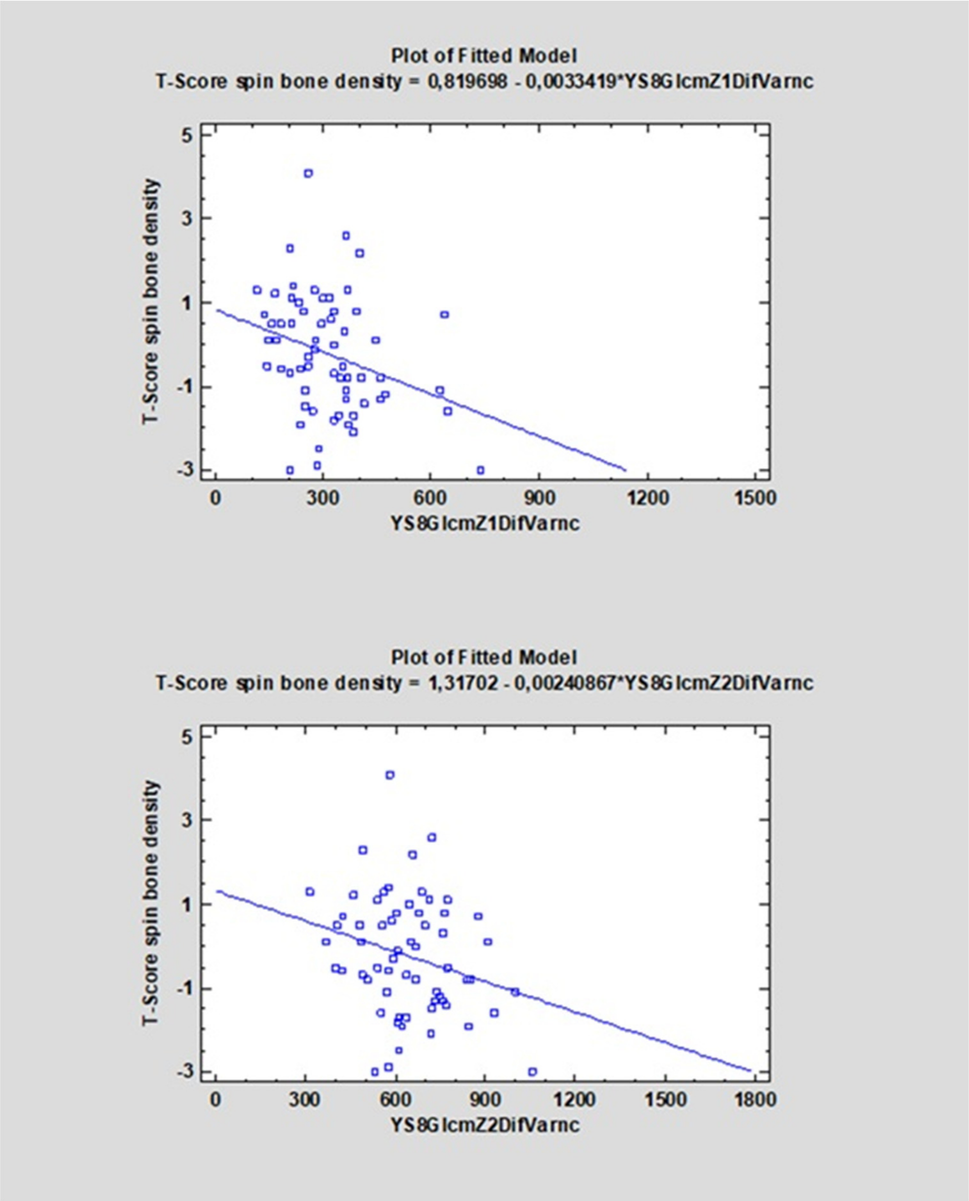

Figure S1. The scatter plot shows the relationship between the variable YS8GlcmZ1DifVarnc, YS8GlcmZ2DifVarnc (X-axis) and the T-score of spinal bone mineral density (Y-axis). A linear regression model indicating a negative trend—higher values of YS8GlcmZ1DifVarnc, YS8GlcmZ2DifVarnc are associated with lower T-scores.

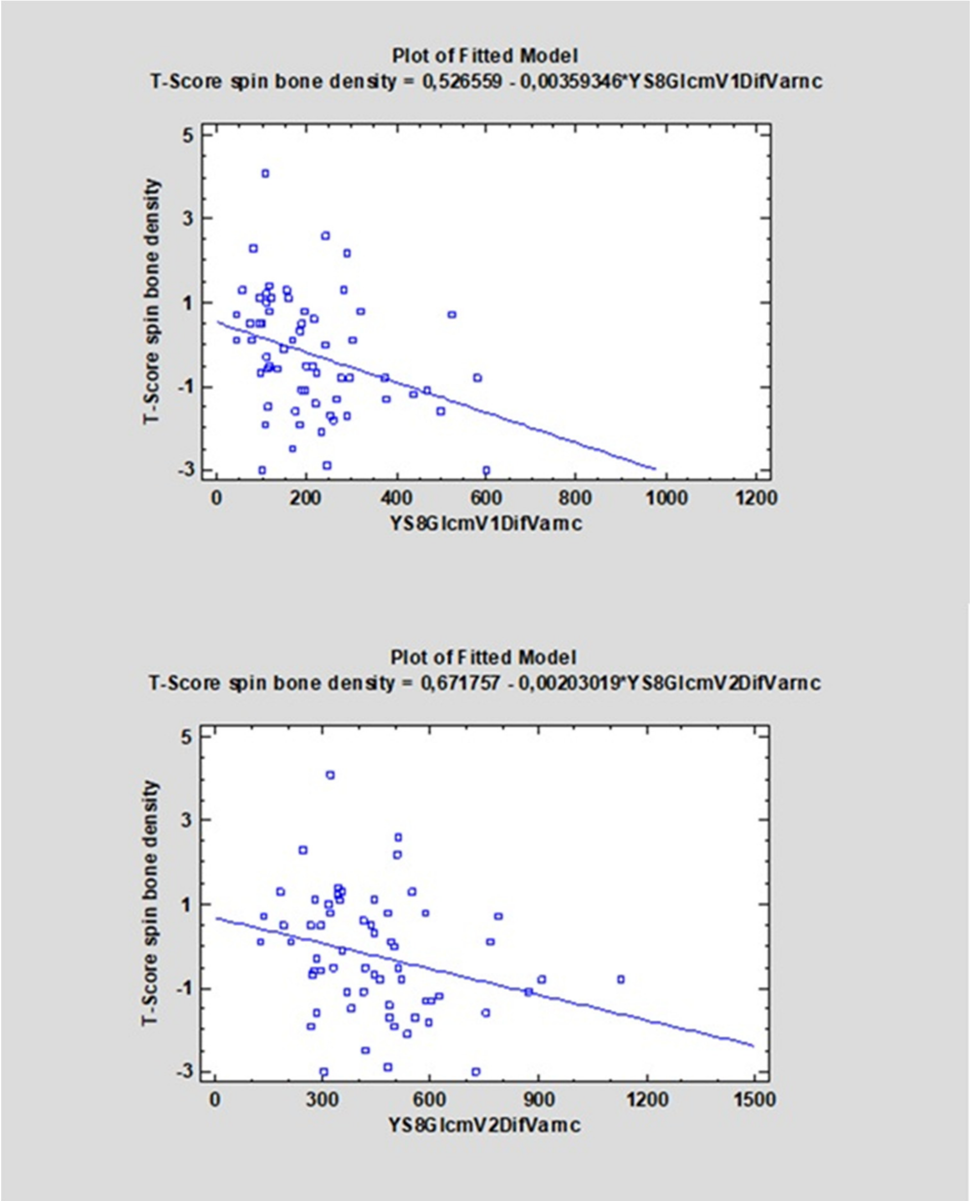

Figure S2. The scatter plot shows the relationship between the variable YS8GlcmV1DifVarnc, YS8GlcmV2DifVarnc (X-axis) and the T-score of spinal bone mineral density (Y-axis). A linear regression model indicating a negative trend—higher values of YS8GlcmV1DifVarnc, YS8GlcmV2DifVarnc are associated with lower T-scores.

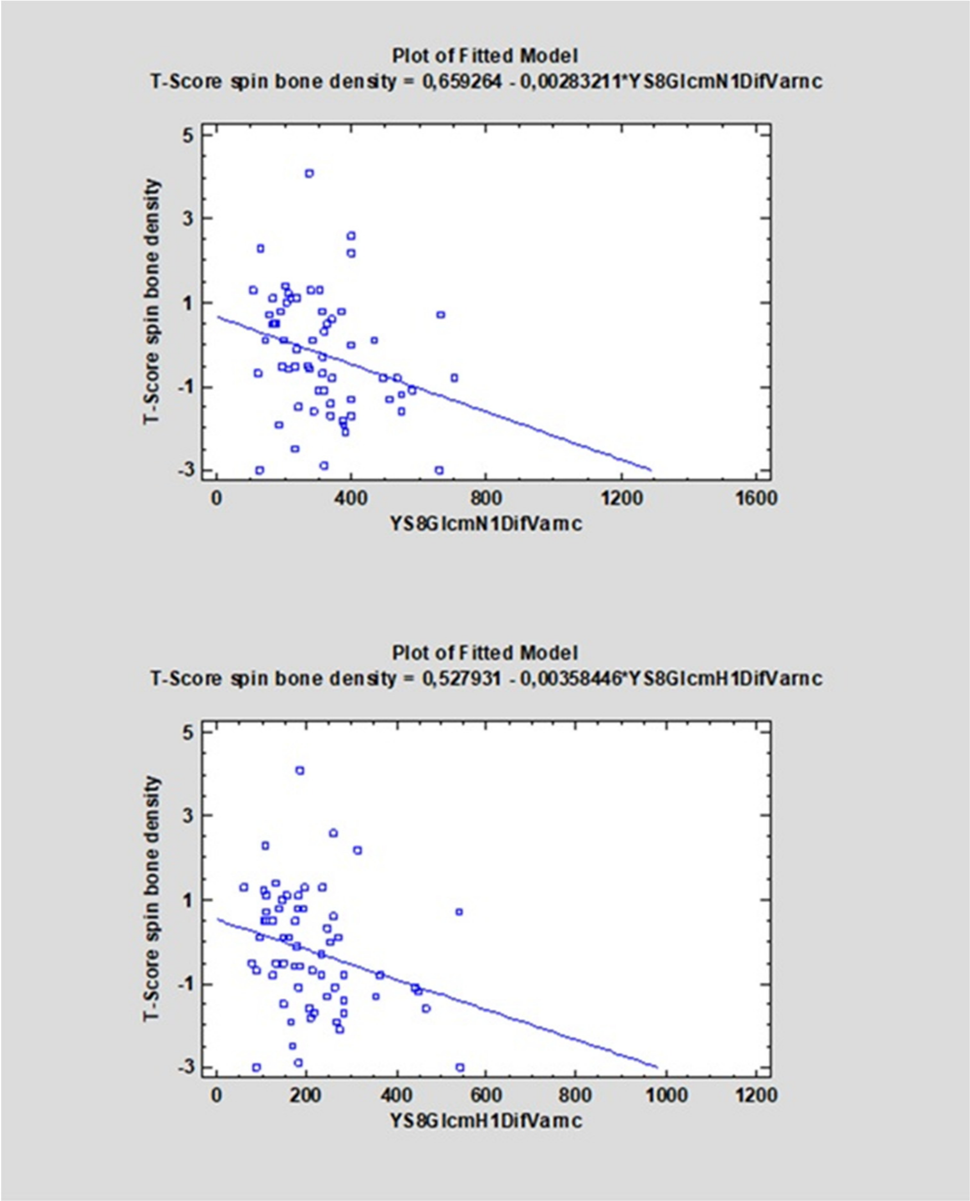

Figure S3. The scatter plot shows the relationship between the variable YS8GlcmN1DifVarnc, YS8GlcmH1DifVarnc (X-axis) and the T-score of spinal bone mineral density (Y-axis). A linear regression model indicating a negative trend—higher values of YS8GlcmN1DifVarnc, YS8GlcmH1DifVarnc are associated with lower T-scores.

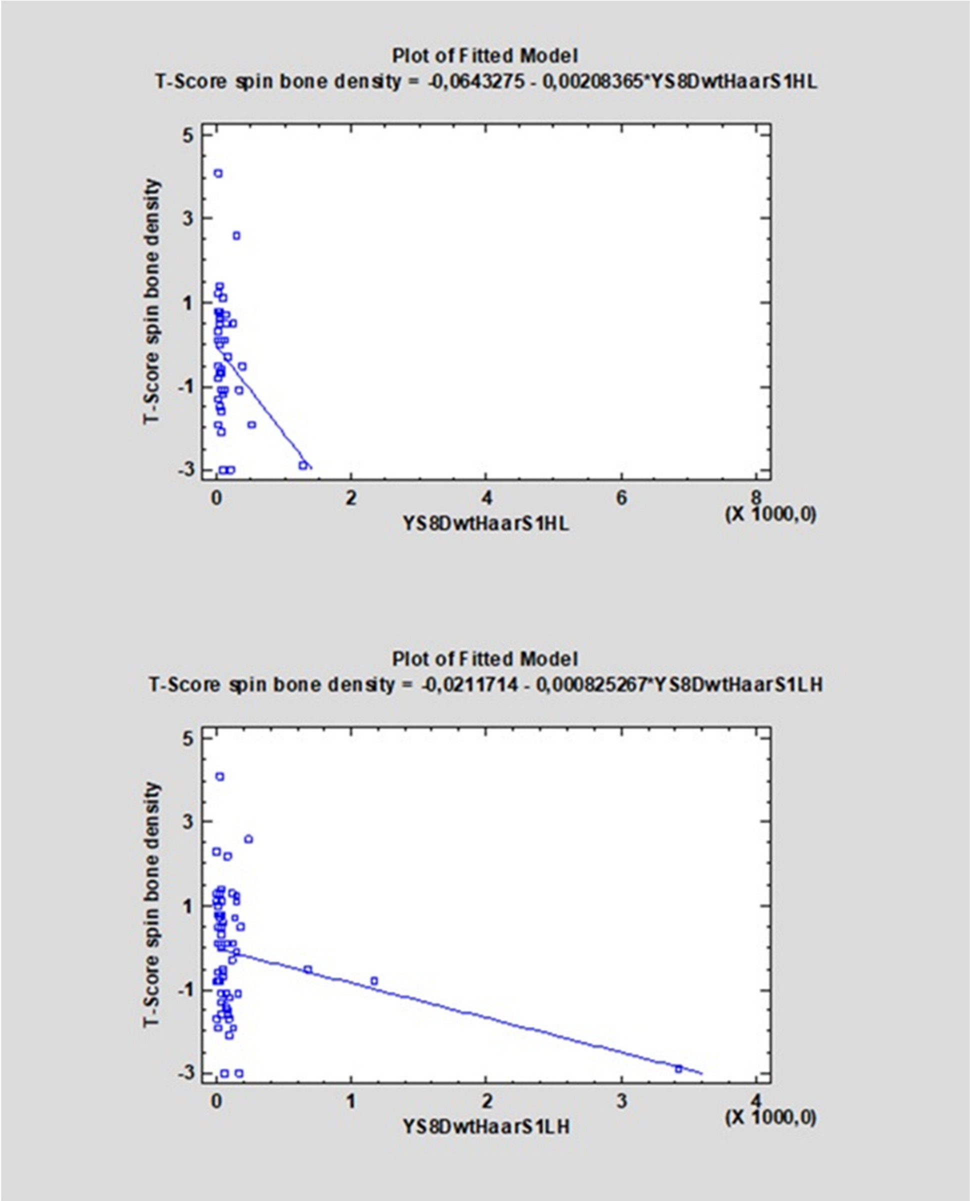

Figure S4. The scatter plot shows the relationship between the variable YS8DwtHaarS1HL, YS8DwtHaarS1LH (X-axis) and the T-score of spinal bone mineral density (Y-axis). A linear regression model indicating a negative trend—higher values of YS8DwtHaarS1HL, YS8DwtHaarS1LH are associated with lower T-scores

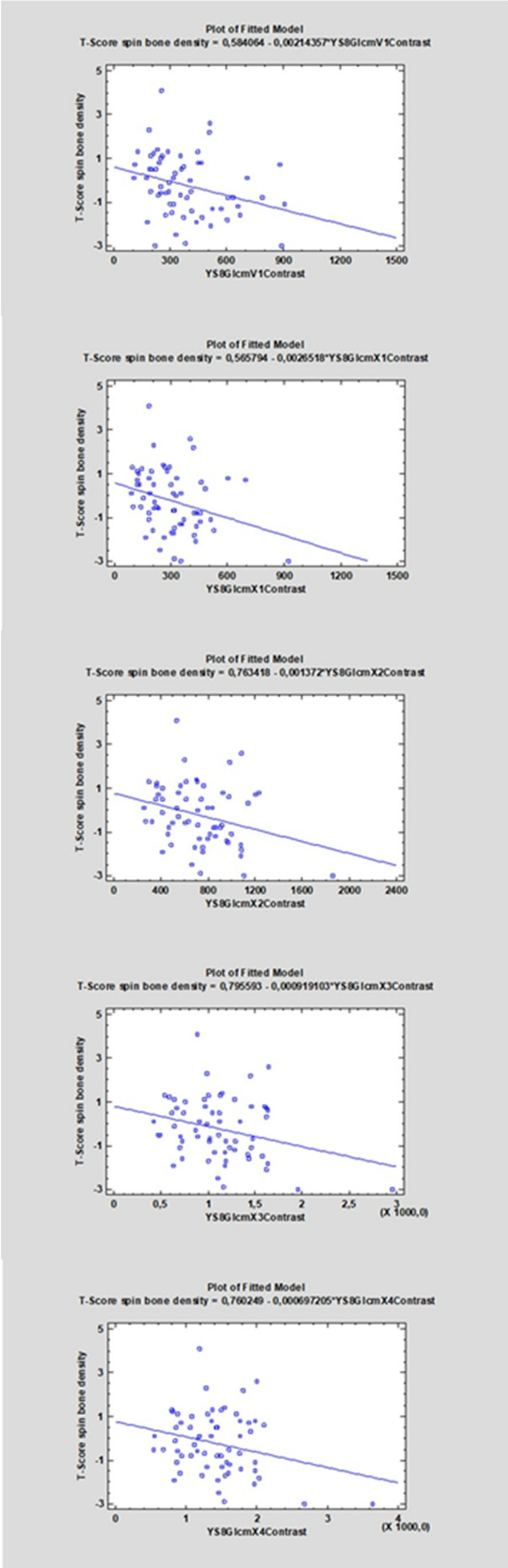

Figure S5. The scatter plot shows the relationship between the variable YS8GlcmV1Contrast, YS8GlcmX1Contrast, YS8GlcmX2Contrast, YS8GlcmX3Contrast, YS8GlcmX4Contrast (X-axis) and the T-score of spinal bone mineral density (Y-axis). A linear regression model indicating a negative

trend—higher values of YS8GlcmV1Contrast, YS8GlcmX1Contrast, YS8GlcmX2Contrast, YS8GlcmX3Contrast, YS8GlcmX4Contrast are associated with lower T-scores

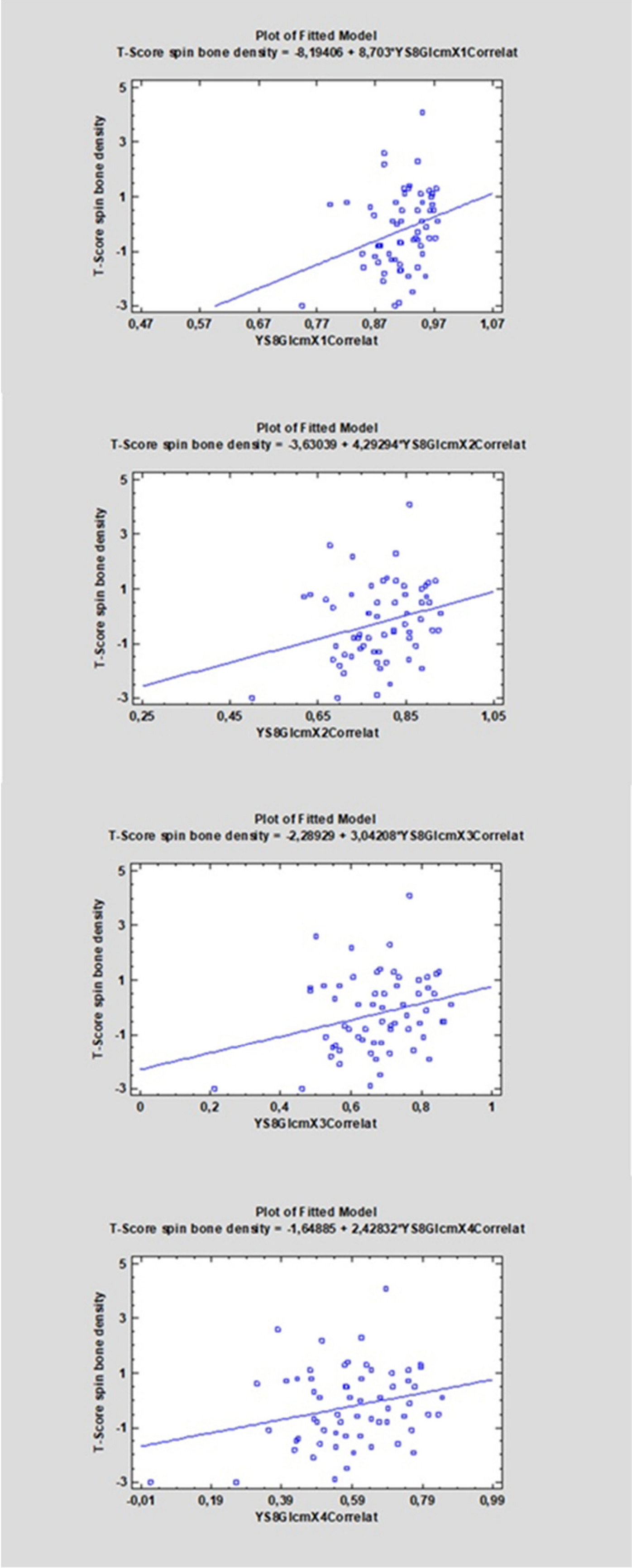

Figure S6. The scatter plot shows the relationship between the variable YS8GlcmX1Correlat, YS8GlcmX2Correlat, YS8GlcmX3Correlat, YS8GlcmX4Correlat (X-axis) and the T-score of spinal

bone mineral density (Y-axis). A linear regression model indicating a positive trend—higher values of YS8GlcmX1Correlat, YS8GlcmX2Correlat, YS8GlcmX3Correlat, YS8GlcmX4Correlat are associated with higher T-scores.

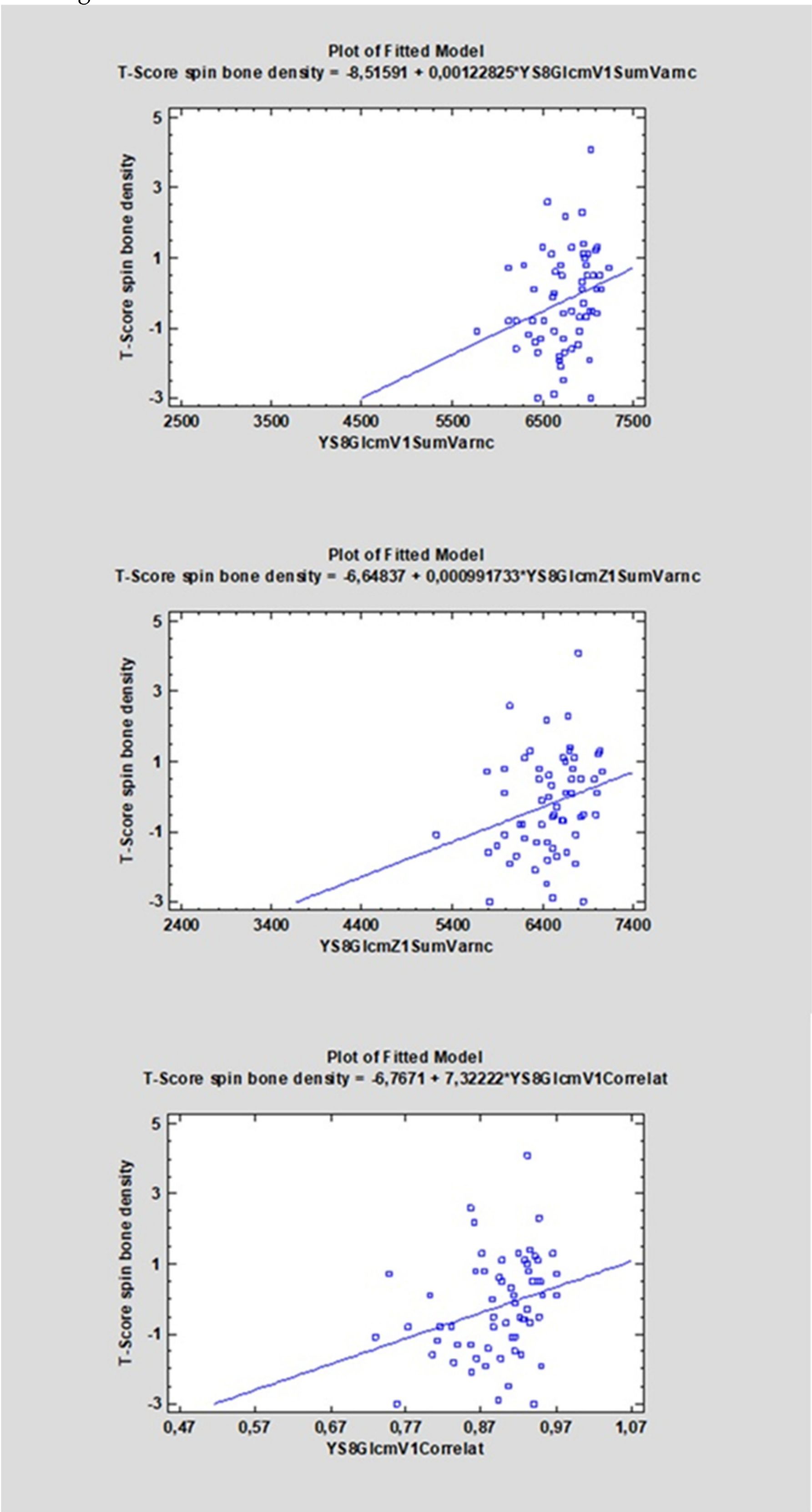

Figure S7. The scatter plot shows the relationship between the variable YS8GlcmV1Correlat, YS8GlcmV1SumVarnc, YS8GlcmZ1SumVarnc (X-axis) and the T-score of spinal bone mineral density

(Y-axis). A linear regression model indicating a positive trend—higher values of YS8GlcmV1Correlat, YS8GlcmV1SumVarnc, YS8GlcmZ1SumVarnc are associated with higher T-scores

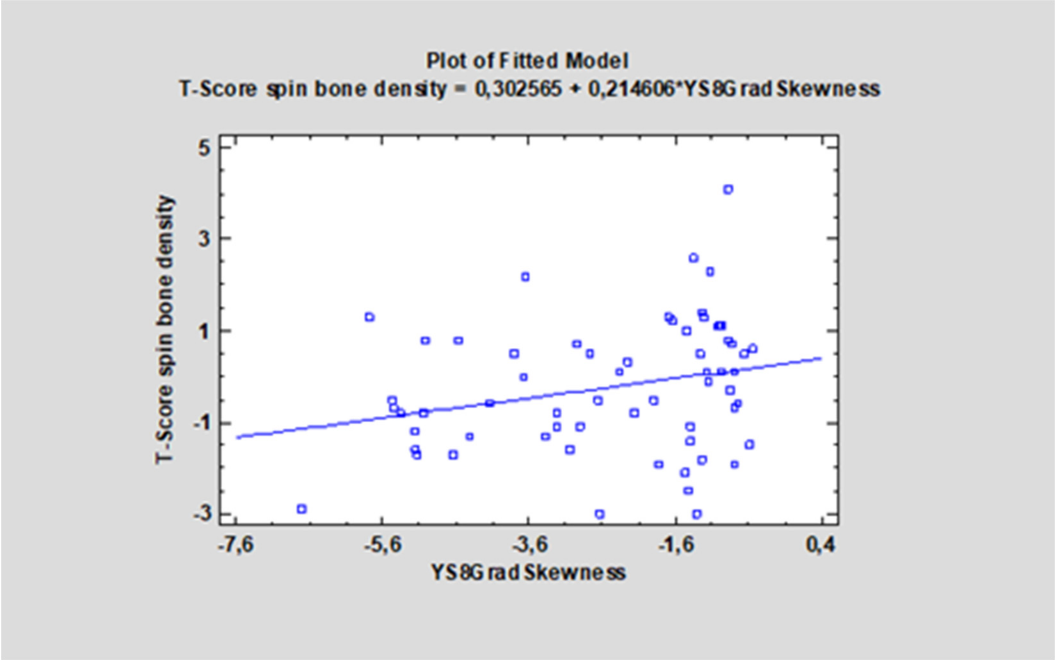

Figure S8. The scatter plot shows the relationship between the variable YS8GradSkewness (X-axis) and the T-score of spinal bone mineral density (Y-axis). A linear regression model indicating a positive trend—higher values of YS8GradSkewness are associated with higher T-scores.

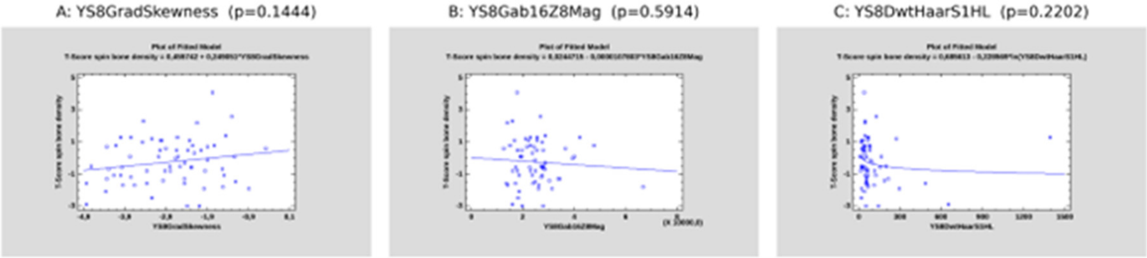

Figure S9. The scatter plot shows the relationship between the variable YS8GradSkewness, YS8Gab16Z8Mag, YS8DwtHaarS1HL (X-axis) and the T-score of spinal bone mineral density (Y-axis). The three presented texture features were not statistically significant when patient-level mean ROI values were analyzed.
